# Self-Assembled Alkylated Polyamine Analogs as Supramolecular Anticancer Agents

**DOI:** 10.3390/molecules27082441

**Published:** 2022-04-10

**Authors:** Diptesh Sil, Sudipta Panja, Chinmay M. Jogdeo, Raj Kumar, Ao Yu, Cassandra E. Holbert, Ling Ding, Jackson R. Foley, Tracy Murray Stewart, Robert A. Casero, David Oupický

**Affiliations:** 1 Department of Pharmaceutical Sciences, Center for Drug Delivery and Nanomedicine, University of Nebraska Medical Center, Omaha, NE 68198, USA; diptesh.sil@unmc.edu (D.S.); sudipta.panja@unmc.edu (S.P.); chinmaymahesh.jogdeo@unmc.edu (C.M.J.); rajkumar.umich@gmail.com (R.K.); ao.yu@beigene.com (A.Y.); ling.ding@unmc.edu (L.D.); 2 Sidney Kimmel Comprehensive Cancer Center, Johns Hopkins School of Medicine, Baltimore, MD 21231, USA; cholber2@jhmi.edu (C.E.H.); jfoley13@jhmi.edu (J.R.F.); tmurray2@jhmi.edu (T.M.S.); rcasero@jhmi.edu (R.A.C.J.)

**Keywords:** polyamine analogs, PG11047, self-assembly, spermine oxidase, cancer therapy

## Abstract

Conformationally restrained polyamine analog PG11047 is a well-known drug candidate that modulates polyamine metabolism and inhibits cancer cell growth in a broad spectrum of cancers. Here, we report a structure–activity relationship study of the PG11047 analogs (HPGs) containing alkyl chains of varying length, while keeping the unsaturated spermine backbone unchanged. Synthesis of higher symmetrical homologues was achieved through a synthetic route with fewer steps than the previous route to PG11047. The amphiphilic HPG analogs underwent self-assembly and formed spherically shaped nanoparticles whose size increased with the hydrophobic alkyl group’s increasing chain length. Assessment of the in vitro anticancer activity showed more than an eight-fold increase in the cancer cell inhibition activity of the analogs with longer alkyl chains compared to PG11047 in human colon cancer cell line HCT116, and a more than ten-fold increase in human lung cancer cell line A549. Evaluation of the inhibition of spermine oxidase (SMOX) showed no activity for PG11047, but activity was observed for its higher symmetrical homologues. Comparison with a reference SMOX inhibitor MDL72527 showed nine-fold better activity for the best performing HPG analog.

## 1. Introduction

Polyamines (spermidine and spermine) and their precursor (putrescine) are natural alkylamines that are absolutely required for both normal and neoplastic cell growth [1]. A wide variety of asymmetric-substituted polyamines have been synthesized and evaluated for their therapeutic efficacy in various types of cancer cells with encouraging results [2,3,4]. Cationic hydrophobic polyamines have been explored as cytotoxic agents and also as gene delivery vectors [5,6]. Among the various polyamines, spermine derivatives showed promising antitumor activity. Molecular simulation studies showed that spermine is bound in a cisoidal conformation, wrapping around the major groove of the double helix of DNA [7]. Hence, the introduction of cisoidal conformation in the spermine structure results in an effective increase in the binding interaction with the DNA or t-RNA [8]. In this context, conformationally restricted analogs of bisethylspermine (BESpm) were synthesized by introducing various restricting moieties, such as cyclopropyl and cyclobutyl, and a double bond in the butyl segment of BESpm. These conformationally restricted polyamine analogs showed an enhanced growth inhibition effect in several human tumor cell lines [8]. PG11047 is an example of a successful polyamine analog with cisoidal conformation and terminal N-ethyl substituents that inhibits the growth of both small (NCI H82 and H69) and non-small (NCI A549 and H157) lung cancer cells in vitro [9], and shows a broad spectrum of anticancer activity in colon cancer cells [10]. PG11047 was found to significantly delay the progression of established tumors in an in vivo A549 xenograft model. PG11047 underwent Phase I and II clinical trials in multiple cancers [1,9,10]. Thus, PG11047 is a promising lead polyamine for further clinical evaluation.

Since the polyamine analogs are well tolerated, and since they exhibit tumor selectivity [11,12], we recently proposed a strategy to improve antitumor responses to these agents by combining them with therapeutic RNA interference (RNAi). Our approach has been based on the idea of prodrugs of polyamine analogs designed to encapsulate and deliver therapeutic RNAs to tumors. We have reported on lipid and polymer prodrugs based either on PG11047 or bisethylnorspermine (BENSpm) [13,14]. The systems were designed to degrade into small molecules that modulate dysregulated polyamine metabolism in cancer to boost the therapeutic RNAi activity. Therapies that combine RNAi with small-molecule drugs have the potential to greatly enhance the treatment repertoire and their efficacy for many types of cancer. Due to the heterogeneity of cancers and the involvement of multiple gene mutations during tumorigenesis and tumor progression, the combination of RNAi with modulators of polyamine metabolism has the potential to provide significant advantages in targeting multiple cancer-associated pathways. The goal of this study was to eliminate the original prodrug approach and replace it with hydrophobized polyamine analogs capable of supramolecular assembly potentially suitable for RNA delivery. Hydrophobic interactions are an important factor causing the self-assembly of amphiphilic molecules in aqueous solution, leading to the rational design and synthesis of various low- and high-molecular-weight amphiphiles with an increase in hydrophobicity towards self-assembly [15]. We thus hypothesized that the higher homologs of PG11047 will eventually self-assemble through non-covalent interactions to form nanoparticles with good biocompatibility, high stability in vitro, and the ability to penetrate intracellular regions in tumor cells and tissues [16]. We report on the synthesis of PG11047 homologs and evaluate their cytotoxic effect and ability to inhibit SMOX.

## 2. Results and Discussion

### 2.1. Synthesis of Alkylated Analogs of PG11047

Earlier synthesis of PG11047 has been reported from *cis*-2-butenediol [8,17]. The diol was acylated with mesitylenesulfonyl chloride to give the activated ester. The ester was then reacted with the sodium salt of *N*^1^-ethylpropane-1,3-diamine dimesitylate. Displacement of the mesitylenesulfonate residues gave tetrasulfonamide. The tetramide was finally deprotected with hydrogen bromide in glacial acetic acid to yield PG11047 as the HBr salt. The HBr salt was finally converted to the hydrochloride salt with HCl gas. This method has several disadvantages. Conversion of the diol to an activated ester (mesitylates) with mesitylenesulfonyl chloride is a low yielding reaction as the mesitylates are often hydrolytically labile. Moreover, the deprotection of the tetrasulfonamides using HBr/AcOH and phenol in the final step has a tedious workup process. The biggest disadvantage of the original methodology is that the process is mostly restricted to the synthesis of PG11047 analogs with ethyl substituents, and is not readily applicable to the synthesis of conformationally restrained polyamines with higher aliphatic chain analogs. Therefore, due to the synthetic complexity of PG11047 [8,17] and other restricted analogs of polyamines, an exhaustive structure activity relationship study based on the lead molecule PG11047 has not yet been reported.

**Scheme 1:**
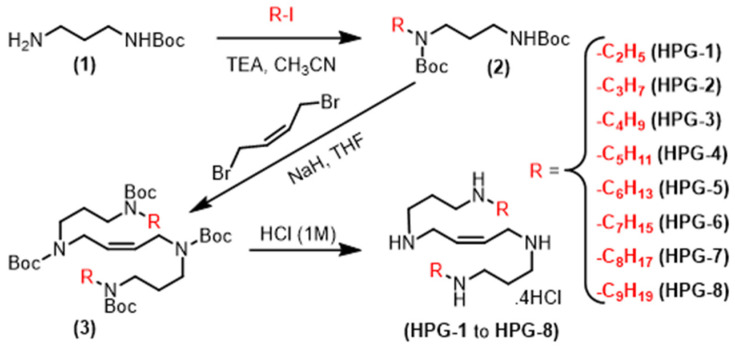
General synthetic scheme to synthesize conformationally restricted HPG analogs.

To overcome this drawback, we developed a new strategy starting from tert-butyl (3-((tert-butoxycarbonyl)amino)propyl)(alkyl)carbamates (**2**). The general synthetic scheme to prepare conformationally restricted alkylated polyamines is presented in Scheme 1. The desired compounds (**2**) were synthesized by *N*-alkylation of tert-butyl (3-aminopropyl)carbamate (**1**) with various alkyl iodides followed by *N*-Boc protection.

The intermediates (**2**) on *N*-alkylation with *cis*-1,4-dibromo-2-butene gave the tetra-Boc polyamine analogs (**3**). Boc-deprotection of these intermediates (**3**) with 1 M HCl in ethyl acetate gave the targeted polyamines as HCl salt. The present methodology used a smaller number of steps than the reported procedure. Replacing the sulfonamido (-SO_2_Mes) with a t-butylcarbamoyl (-Boc) protecting group for amines yielded the conformationally restrained polyamines as the HCl salt during final deprotection without the use of corrosive reagents, such as HBr, phenol, and HCl gas. The synthesized analogs were characterized by ^1^H-, ^13^C-NMR, and mass spectroscopic (Orbitrap fusion, Thermo Fischer Scientific, Waltham, MA, USA) analysis.

### 2.2. Self-Assembly

Self-assembly processes of amphiphilic molecules involving supramolecular chemistry provide new opportunities for designing novel materials for advanced applications in colloid, polymer, and materials science, thus playing a key role in bionanotechnology. Separation of hydrophilic and hydrophobic segments of amphiphiles represents the primary driving force for the formation of self-assembled structures such as micelles, vesicles, and bilayers [18]. The non-covalent interactions of the synthesized HPG analogs resulted in the formation of nanoparticles of spherical morphology through the self-assembly process in water. It is reasonable to consider that these nanoparticles in an aqueous solution consist of a hydrophobic core represented by the long alkylated chain of the HPG analogs and the hydrophilic shell represented by the unsaturated polyamine (*cis* spermine). The self-assembly was expected to produce particles with a large number of positive charges that could later be used to bind with nucleic acids, and these materials could then be explored in the delivery of nucleic acids such as siRNA and mRNA.

**Figure 1:**
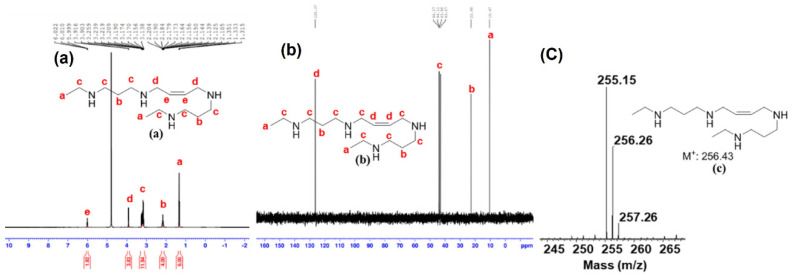
(**a**) ^1^H NMR, (**b**) ^13^C NMR, and (**c**) mass spectrometric analysis (Orbitrap fusion) of PG11047 (HPG-1).

**Figure 2:**
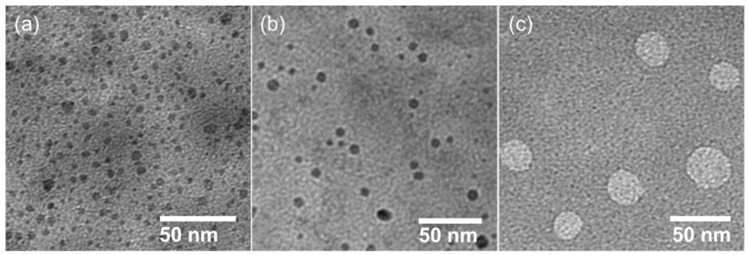
Representative TEM images of nanoparticles formed by the self-assembly of HPG-4 (**a**), HPG-6 (**b**), and HPG-7 (**c**).

The hydrodynamic particle size of the self-assembled HPG analogs was characterized by using dynamic light scattering (DLS). The size of all the formed particles ranged between 167 and 626 nm (Table 1), depending on the chain length of the HPG alkyl groups (Figure 1). The results showed an increase in particle size with the increasing chain length of the hydrophobic alkyl groups of the HPG analogs. For example, HPG2 with the shortest alkyl chain showed a size around 173 nm, whereas HPG-8 with the longest alkyl chain (-C_9_H_19_) showed a size around 626 nm. Furthermore, bulk morphology of the self-assembled particles was captured under TEM microscopy. Particles appeared as spherical in shape, with sizes smaller than the sizes obtained from DLS due to hydration. For example, the DLS sizes (Table 1) of HPG-4, HPG-6, and HPG-7 were 174 nm, 225 nm, and 247 nm, respectively, whereas the average sizes obtained from TEM (Figure 2) were 6 nm, 8 nm, and 30 nm, respectively.

### 2.3. Inhibition of the Cancer Cell Growth and SMOX by the HPG Nanoparticles

Cellular dose–response studies were performed using the Promega CellTiter-Blue Viability assay (Madison, WI, USA). All the HPG analogs were first screened for antiproliferative activity against human colon cancer cell line HCT116. The analogs HPG-8 and HPG-2 showed promising inhibitory effects against HCT116 with IC_50_ < 1 µM. Moreover, compounds HPG-6 and HPG-7 showed cytotoxicity in HCT116 cells at a concentration of IC_50_ values of 3 µm and 1 µM, respectively. On the other hand, the original compound PG11047 had an IC_50_ of 8.0 µM against the growth of HCT116 cells (Table 2). Thus, the higher HPG homologs were found to be at least eight-fold more active than PG11047.

All the HPG analogs were also screened for antiproliferative activity against the human lung adenocarcinoma line A549. Analogs HPG-2 and HPG-7 showed promising inhibitory effects against A549 cells with IC_50_ < 1µM.

Moreover, compounds HPG-5, HPG-6, and HPG-8 showed cytotoxicity in A549 cells at a concentration of IC_50_ values of 7 µM, 3 µM, and 7 µM, respectively. The original compound PG11047 had an IC_50_ > 10.0 µM in A549 cells (Table 2).

The induction of SMOX has been implicated in the etiology of several epithelial cancers associated with infection and/or inflammation, including lung, colon, gastric, skin, and prostate cancers [19,20]. As Spm is a substrate of SMOX [21], it was of interest to explore the conformationally restrained HPG analogs as SMOX inhibitors. Thus, all the HPG analogs were screened for the inhibition of SMOX with respect to the established irreversible inhibitor MDL72527 with an IC_50_ value of 90 µM [21]. We examined the ability of the HPG analogs to directly inhibit the SMOX enzyme activity. Our initial screening of the HPG analogs suggests that higher homologues may be potent SMOX inhibitors and serve as lead compounds for further development. Higher symmetrical analogs of PG11047 with bis hexyl, heptyl, octyl, and nonyl (HPG-5, HPG-6, HPG-7, and HPG-8) analogs were found to have an IC_50_ value of 90 µM, 30 µM, <10 µM, and <10 µM, respectively. Analogs HPG-7 and HPG-8 were thus around nine-fold more active than MDL 72,527 (IC_50_~90µM) (Table 2).

## 3. Materials and Methods

### 3.1. Materials

*N*-Boc-1,3-propanediamine was purchased from aa Blocks (San Diego, CA, USA). *Cis*-1,4-dibromo-2-Butene was obtained from eNovation Chemicals Product List (Bridgewater Township, NJ, USA). All alkyl iodides were purchased from Sigma-Aldrich (Saint Louis, MO, USA). Di-t-butyl dicarbonate was purchased from Oakwood Chemical (Estill, SC, USA). Triethylamine and sodium hydride were purchased from Sigma-Aldrich. HCl (1 M solution in ethyl acetate) was purchased from Acros Organics (Waltham, MA, USA). All the solvents and reagents used were obtained from Sigma-Aldrich as such, unless noted otherwise.

### 3.2. General Procedure for the Synthesis and Characterization of (Z)-N^1^,N^1′^-(But-2-Ene-1,4-Diyl)Bis(N^3^-Ethylpropane-1,3-Diamine, (PG11047)

Next, 575 mg (3.3 mmol) of tert-butyl (3-aminopropyl)carbamate (**1**), 318.4 µL (3.96 mmol) of ethyl iodide, and 551 µL of triethylamine (3.96 mmol) were added to 20 mL of dry acetonitrile. The reaction was heated to 75 °C for 16–18 h. After completion of the reaction, 1.8 g of Di-t-butyl dicarbonate (8.24 mmol) and 551 µL (3.96 mmol) of triethylamine were further added to the same reaction pot and stirred for 4 h. The completion of the reaction was monitored by TLC. Acetonitrile was removed under reduced pressure to yield a crude viscous oil. The crude mass was purified through silica gel column chromatography using a hexane-ethyl acetate (60:40) system to yield t-butyl (3-((tert-butoxycarbonyl)amino)propyl)(ethyl)carbamate (**2a**) as colorless viscous oil (598 mg, yield—60%). To 398.2 mg (1.316 mmol) of the intermediate (**2a**), 211 mg of sodium hydride (5.264 mmol, 60% dispersion in mineral oil) was added in 15 mL of dry THF at 0 °C and stirred for 45 min. After 45 min, 74 µL (0.627 mmol) of *cis*-1,4-dibromobut-2-ene was added at 0 °C and the reaction was slowly brought to room temperature. The reaction was thereafter heated at 45 °C for 24 h. After its completion, THF was removed under reduced pressure to yield a crude yellow solid mass. To this solid mass, ice water was slowly added to quench excess sodium hydride. The reaction mass was then extracted three times, each with 50 mL of ethyl acetate. The combined organic extracts were removed under reduced pressure to yield pale yellow viscous oil di-tert-butyl ((2,2,13,13-tetramethyl-4,11-dioxo-3,12-dioxa-5,10-diazatetradec-7-ene-5,10-diyl)bis(propane-3,1-diyl))(*Z*)-bis(ethylcarbamate) (**3a**). The crude intermediate (**3a**) was purified through silica gel column chromatography using a hexane-ethyl acetate (50:50) system to yield pure intermediate (**3a**) as a colorless oil (458 mg, yield—53%). To 458 mg of the pure intermediate (**3a**, 0.221 mmol), 8 mL of 1 M HCl in ethyl acetate was added and stirred for 8 h in a sealed environment. Ethyl acetate was removed under reduced pressure to yield the targeted molecule (*Z*)-*N*^1^,*N*^1′^-(but-2-ene-1,4-diyl)bis(*N*^3^-ethylpropane-1,3-diamine) (PG11047) as an amorphous white hydrochloride salt (Scheme 1). The structure was further confirmed by ^1^H, ^13^C NMR and mass spectroscopic analysis (yield—53% (280 mg)). ^1^H NMR (D_2_O, 400 MHz): δ 6.02–6.00 (*t*, *J* = 4.4 Hz, 2 H), 3.92–3.90 (m, 4 H), 3.26–3.14 (m, 12 H), 2.20–2.11 (m, 4 H), and 1.35–1.32 (*t*, *J* = 7.2 Hz, 6 H) ppm. ^13^C NMR (400 MHz, D_2_O): δ 126.37, 44.17, 44.11, 43.80, 43.07, 22.80, and 10.47 ppm. MS (Orbitrap) was calculated for C_14_H_32_N_4_, *m*/*z* 256.26, found 256.26 [M^+^] and 257.26 [M + H]^+^ (Figure 1).

Spectroscopic data for all the HPG analogs (HPG-2 to HPG-8) are described in Supplementary Materials (Figures S1–S25). Solvents were removed under reduced pressure using standard rotary evaporators. Flash column chromatography was carried out using Biotage^®^ Sfär silica HC columns (Uppsala, Sweden) of 10 g (20 µm) on Biotage Selekt instrument unless otherwise mentioned. Thin-layer chromatography was carried out on silica gel CCM precoated aluminum sheets. An accurate Agilent ESI-TOF mass spectrometer (Santa Clara, CA, USA) (mass accuracy of 5 ppm), operating in the positive ion acquisition mode, was used. The total ion current from 150 to 3500 Da was measured. The molecular weight of the compounds was determined using Bruker MALDI-TOF/TOF autoflex^®^ maX (Billerica, MA, USA) and Thermo Scientific^TM^ (Waltham, MA, USA) Orbitrap Fusion^TM^. ^1^H and ^13^C NMR were determined using a Bruker (Billerica, MA, USA) NMR spectrometer and analyzed using Bruker TOPSPIN software 3.6.4 (Billerica, MA, USA).

Synthesis of (*Z*)-*N*^1^,*N*^1′^-(but-2-ene-1,4-diyl)bis(*N*^3^-propylpropane-1,3-diamine) (HPG-2): (yield—52%). ^1^H NMR (400 MHz, D_2_O): δ 5.99–5.98 (m, 2 H), 3.89–3.88 (m, 4 H), 3.23–3.15 (m, 8 H), 3.07–3.04 (m, 4 H), 2.19–2.11 (m, 4 H), 1.77–1.68 (m, 4 H), and 1.01–0.98 (*t*, *J* = 7.2 Hz, 6 H) ppm. ^13^C NMR (400 MHz, D_2_O): δ 126.35, 49.38, 44.22, 44.16, 44.11, 22.75, 19.13, and 10.11 ppm. MS (Orbitrap) calculated for C_16_H_36_N_4_, *m*/*z* 284.29, found 284.23 [M^+^].
Synthesis of (*Z*)-*N*^1^,*N*^1′^-(but-2-ene-1,4-diyl)bis(*N*^3^-butylpropane-1,3-diamine) (HPG-3): (yield—50%). ^1^H NMR (400 MHz, D_2_O): δ 6.03–5.95 (m, 2 H), 3.89–3.88 (m, 4 H), 3.26–3.15 (m, 8 H), 3.11–3.04 (m, 4 H), 2.19–2.11 (m, 4 H), 1.72–1.65 (m, 4 H), 1.46–1.37 (m, 4 H), and 0.97–0.93 (*t*, *J* = 7.2 Hz, 6 H) ppm. ^13^C NMR (400 MHz, D_2_O): δ 126.35, 47.61, 44.23, 44.16, 44.11, 27.50, 22.76, 19.10, and 12.70 ppm. MS (Orbitrap) calculated for C_18_H_40_N_4_, *m*/*z* 312.32, found 313.25 [M + H]^+^.
Synthesis of (*Z*)-*N*^1^,*N*^1′^-(but-2-ene-1,4-diyl)bis(*N*^3^-pentylpropane-1,3-diamine) (HPG-4): (yield—50%). ^1^H NMR (400 MHz, D_2_O): δ 6.00–5.98 (*t*, *J* = 4.4 Hz, 2 H), 3.89–3.80 (m, 4 H), 3.23–3.15 (m, 8 H), 3.11–3.07 (m, 4 H), 2.19–2.11 (m, 4 H), 1.75–1.67 (m, 4 H), 1.39–1.35 (m, 8 H), and 0.93–0.89 (m, 6 H) ppm. ^13^C NMR (400 MHz, D_2_O): δ 126.36, 47.85, 44.21, 44.17, 44.11, 27.78, 25.14, 22.75, 21.43, and 12.98 ppm. MS (Orbitrap) calculated for C_20_H_44_N_4_, *m*/*z* 340.35, found *m*/*z* 341.36 [M + H]^+^ and 171.18 [C_10_H_23_N_2_]^+^.
Synthesis of (*Z*)-*N*^1^,*N*^1′^-(but-2-ene-1,4-diyl)bis(*N*^3^-hexylpropane-1,3-diamine) (HPG-5): (yield—53%). ^1^H NMR (400 MHz, D_2_O): δ 5.99–5.98 (m, 2 H), 3.89–3.88 (m, 4 H), 3.23–3.15 (m, 8 H), 3.10–3.07 (m, 4 H), 2.19–2.11 (m, 4 H), 1.74–1.67 (m, 4 H), 1.40–1.32 (m, 12 H), and 0.91–0.88 (m, 6 H). ^13^C NMR (400 MHz, D_2_O): δ 126.36, 47.88, 44.21, 44.17, 44.12, 30.40, 25.41, 25.30, 22.75, 21.68, and 13.18 ppm. MS (Orbitrap) calculated for C_22_H_48_N_4_, *m*/*z* 368.38, found *m*/*z* 369.39 [M + H]^+^ and 185.20 [C_11_H_25_N_2_]^+^.
Synthesis of (*Z*)-*N*^1^,*N*^1′^-(but-2-ene-1,4-diyl)bis(*N*^3^-heptylpropane-1,3-diamine) (HPG-6): (yield—50%). ^1^H NMR (400 MHz, D_2_O): δ 5.99 (s, 2 H), 3.89–3.81 (m, 4 H), 3.23–3.15 (m, 8 H), 3.10–3.07 (m, 4 H), 2.19–2.11 (m, 4 H), 1.72–1.67 (m, 4 H), 1.37–1.31 (m, 16 H), and 0.90–0.87 (m, 6 H) ppm. ^13^C NMR (400 MHz, D_2_O): δ 126.36, 47.88, 44.21, 44.17, 44.12, 30.75, 27.83, 25.58, 25.46, 22.76, 21.86, and 13.30 ppm. MS (Orbitrap) calculated for C_24_H_52_N_4_, *m*/*z* 396.41, found *m*/*z* 397.43 [M + H]^+^ and 199.21 [C_12_H_27_N_2_]^+^.
Synthesis of (*Z*)-*N*^1^,*N*^1′^-(but-2-ene-1,4-diyl)bis(*N*^3^-octylpropane-1,3-diamine) (HPG-7): (yield—51%). ^1^H NMR (400 MHz, D_2_O): δ 5.98–5.96 (*t*, *J* = 3.6 Hz, 2 H), 3.87–3.86 (m, 4 H), 3.21–3.13 (m, 8 H), 3.08–3.05 (m, 4 H), 2.16–2.09 (m, 4 H), 1.72–1.66 (m, 4 H), 1.39–1.28 (m, 20 H), and 0.88–0.85 (*t*, *J* = 5.6 Hz, 6 H) ppm. ^13^C NMR (400 MHz, D_2_O): δ 126.36, 47.88, 44.21, 44.17, 44.11, 30.97, 28.12, 28.10, 25.62, 25.45, 22.76, 21.95, and 13.35 ppm. MS (Orbitrap) calculated for C_26_H_56_N_4_, *m*/*z* 424.45, found *m*/*z* 425.45 [M + H]^+^ and 213.23 [C_13_H_29_N_2_]^+^.
Synthesis of (*Z*)-*N*^1^,*N*^1′^-(but-2-ene-1,4-diyl)bis(*N*^3^-nonylpropane-1,3-diamine) (HPG-8): (yield—51%). ^1^H NMR (400 MHz, D_2_O): δ 5.98 (s, 2 H), 3.89–3.80 (m, 4 H), 3.23–3.15 (m, 8 H), 3.10–3.06 (m, 4 H), 2.18–2.11 (m, 4 H), 1.72–1.67 (m, 4 H), 1.36–1.30 (m, 24 H), and 0.88–0.87 (m, 6 H) ppm. ^13^C NMR (400 MHz, D_2_O): δ 126.36, 47.89, 44.21, 44.17, 44.11, 31.08, 28.40, 28.31, 28.14, 25.61, 25.46, 22.76, 22.00, and 13.38 ppm. MS (MALDI) calculated for C_28_H_60_N_4_, *m*/*z* 452.48, found 453.41 [M + H]^+^.

### 3.3. Measurement of Size and Zeta Potential of the Self-Assembled Nanoparticles

To achieve self-assembly, each sample was dispersed in aqueous solution under sonication (Branson Ultrasonic, working frequency 40 Hz at room temperature) for 30 min. Thereafter, the sample was allowed to undergo self-assembly by keeping it undisturbed for another 30 min. The size and surface zeta potential of the self-assembled nanoparticles were analyzed using a Brookhaven NanoBrook (Holtsville, NY, USA) Omni particle size and zeta potential analyzer, respectively.

### 3.4. TEM Imaging

A 10 µL volume of nanoparticle dispersion was meticulously placed on a plasma-treated copper grid (carbon-coated). After 5 min, the excess volume of nanoparticles was absorbed by a regular filter paper. The grid was then stained with 2% phosphotungstic acid (6 µL) for 1 min, and, similarly, the excess staining solution was absorbed by a regular filter paper. The grid was then allowed to dry in air for another 5 min. The image was captured under an FEI-Tecnai G2 transmission electron microscope (Hillsboro, OR, USA) at 80 kV, equipped with an AMT digital imaging system (Woburn, MA, USA).

### 3.5. Cell Lines and Culture Conditions

The human non-small cell lung carcinoma cell lines A549 (ATCC CCL-185) were maintained in RPMI 1640 medium containing 9% bovine calf serum, penicillin, and streptomycin at 37 °C, 5% CO_2_. For all experiments, cells were seeded and allowed to attach overnight. The medium was then aspirated and replaced with that containing polyamine PG analogs at the indicated concentrations. Cells were incubated for the times indicated at 37 °C, 5% CO_2_. The HCT 116 (ATCC CCL-247) human colorectal carcinoma cell lines were grown in McCoy’s 5 A medium (Thermo Fischer Scientific, Waltham, MA, USA), supplemented with 10% FBS at 37 °C with 5% CO_2_ in a humidified chamber.

### 3.6. SMOX Activity Assay and Enzyme Inhibition Studies

SMOX activity was measured using a chemiluminescent enzyme-based assay, detecting the formation of H_2_O_2_ in the presence of HPG analogs as the substrate, as described [21]. To measure the activity of these polyamines against SMOX, the enzyme (300 ng) in 0.083 M glycine buffer (pH 8.0) and the inhibitor (0–250 µM) were added to the luminol-HRP master mix and incubated at 37 °C for 2 min. PG was then added to the reaction mixture at a final concentration of 250 µM, vortexed for 3 s, and chemiluminescence was integrated over 40 s. Data were averaged and normalized to the blank reaction (no inhibitor) as % SMOX activity. Inactivated SMOX served as a negative control and was accounted for in the calculations.

### 3.7. Cytotoxicity of the PG Analogs In Vitro

The cytotoxicity of the HPG analogs was evaluated in HCT116 and A549 cells by the Promega CellTiter Blue Cell viability assay [22]. The cells were seeded in 96-well plates at a density of four thousand cells/well 24 h before the polyamine treatment. The original medium was replaced with serial dilutions of the PG analogs in a cell culture medium and incubated for another 24 h. The mixture of 100 μL cell culture medium and 20 μL CellTiter Blue reagent was added into each well after the removal of the media with polyamines. After 2 h of incubation, the fluorescence intensity [FI] was measured at λex/λem = 560/590 nm by a microplate reader (Molecular Devices, San Jose, CA, USA). The relative cell viability (%) was calculated as [FI]treated/[FI]untreated × 100. Polyamine concentration, causing a 50% decrease in the cell viability, was defined as IC_50_ and auto-calculated in GraphPad Prism 9.0.0 (Prism 9, San Diego, CA, USA) with the dose–response analysis.

## 4. Conclusions

PG11047 analogs with varying terminal *N*-alkyl substituents (C_2_–C_9_) were synthesized and evaluated for cytotoxicity in HCT116 and A549 cell lines. Higher analogs, including HPG-7 and HPG-8, owing to their greater hydrophobicity, improved the cytotoxicity in the tested cancer cell lines when compared with PG11047. These higher analogs were also found to be more active than the reference compound MDL72527 as potent SMOX inhibitors.

## Figures and Tables

**Table 1:** Hydrodynamic size and zeta potential of self-assembled HPG analogs.

Sample	Particle Size (nm)	PDI	Zeta Potential (mV)
HPG-2	173 ± 7	0.21	27
HPG-3	186 ± 3	0.064	24
HPG-4	174 ± 3	0.15	15
HPG-5	158 ± 4	0.23	16
HPG-6	225 ± 6	0.17	22
HPG-7	318 ± 8	0.13	11
HPG-8	626 ± 83	0.17	17

**Table 2:** Evaluation of inhibition of SMOX, HCT116, and A549 cell lines.

Sample Code	IC_50_ HCT116	IC_50_ A549	IC_50_ SMOX *
PG11047	8 µM	>10 µM	ND
HPG-2	<1.0 µM	<1.0 µM	ND
HPG-3	8 µM	>10 µM	ND
HPG-4	>10 µM	10 µM	ND
HPG-5	8 µM	7 µM	90 µM
HPG-6	3 µM	3 µM	30 µM
HPG-7	1.0 µM	<1.0 µM	<10 µM
HPG-8	<1.0 µM	7 µM	<10 µM

Effects of HPG on cell growth and SMOX activity. Cellular dose–response studies were performed using the Promega CellTiter Blue viability assay. ND—not determined. The SMOX assay was performed using purified recombinant human SMOX protein and luminol-based assay as previously reported. * Note that the most used inhibitor of SMOX has an approximate IC50 of 80 µM against purified enzyme.

## Data Availability

The data presented in this study are available in Supplementary Materials.

## Supplementary Materials

The following supporting information can be downloaded at: https://www.mdpi.com/article/10.3390/molecules27082441/s1, The supporting information contains the ^1^H, ^13^C, and mass spectroscopy data of the polyamine analogs (from HPG-2 to HPG-8).

## Sample Availability

Samples of the compounds are available from the authors.
