# Supplementary material for: Self-Assembled Alkylated Polyamine Analogs as Supramolecular Anticancer Agents
Source: Molecules. 2022 Apr 10;27(8):2441. doi: 10.3390/molecules27082441 (PMC9032695; doi:10.3390/molecules27082441)
Supplement: Supplementary file 1 [file molecules-27-02441-s001.zip › molecules-1654178-supplementary.pdf]

Supporting information for "Self-assembled alkylated polyamine analogs as supramolecular anticancer agents"

Diptesh Sil[1], Sudipta Panja[1], Chinmay M. Jogdeo[1], Raj Kumar[1], Ao Yu[1], Cassandra E. Holbert[2], Ling Ding[1], Jack-son R. Foley[2], Tracy Murray Stewart[2], Robert A. Casero, Jr.[2], David Oupický[1,*]

[1]Department of Pharmaceutical Sciences, Center for Drug Delivery and Nanomedicine, University of Nebraska Medical Center, Omaha, NE 68198, USA

[2]Sidney Kimmel Comprehensive Cancer Center, Johns Hopkins School of Medicine, Baltimore, MD 21231, USA

*Correspondence: david.oupicky@unmc.edu.

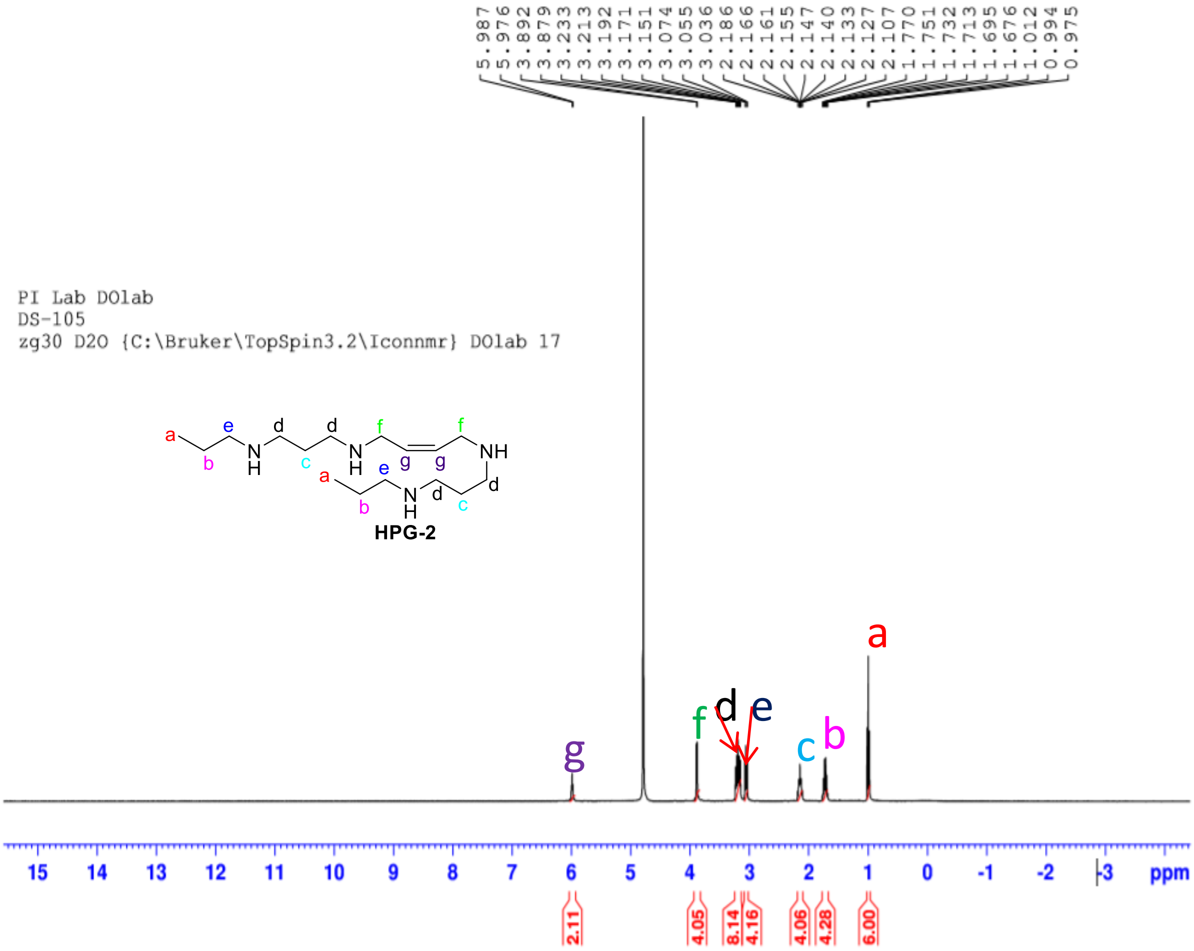

Figure S1. ^{1}H NMR with C_3 side chain (HPG-2)

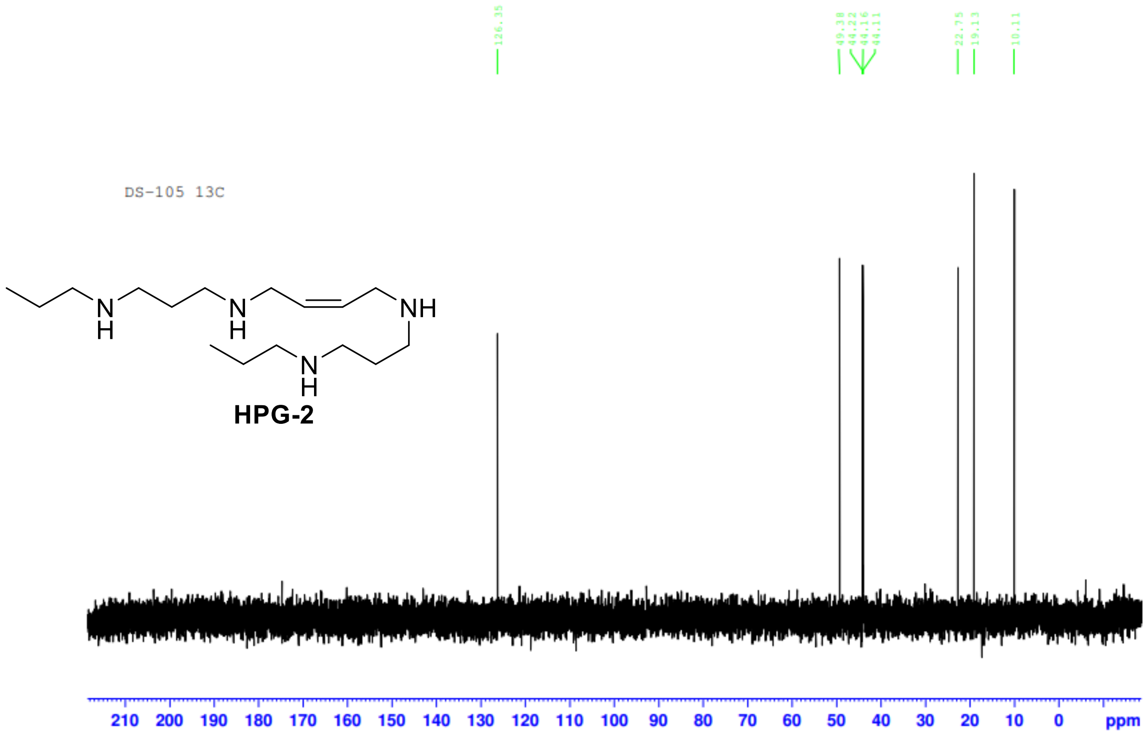

Figure S2. ^{13}C NMR with C_3 side chain (HPG-2)

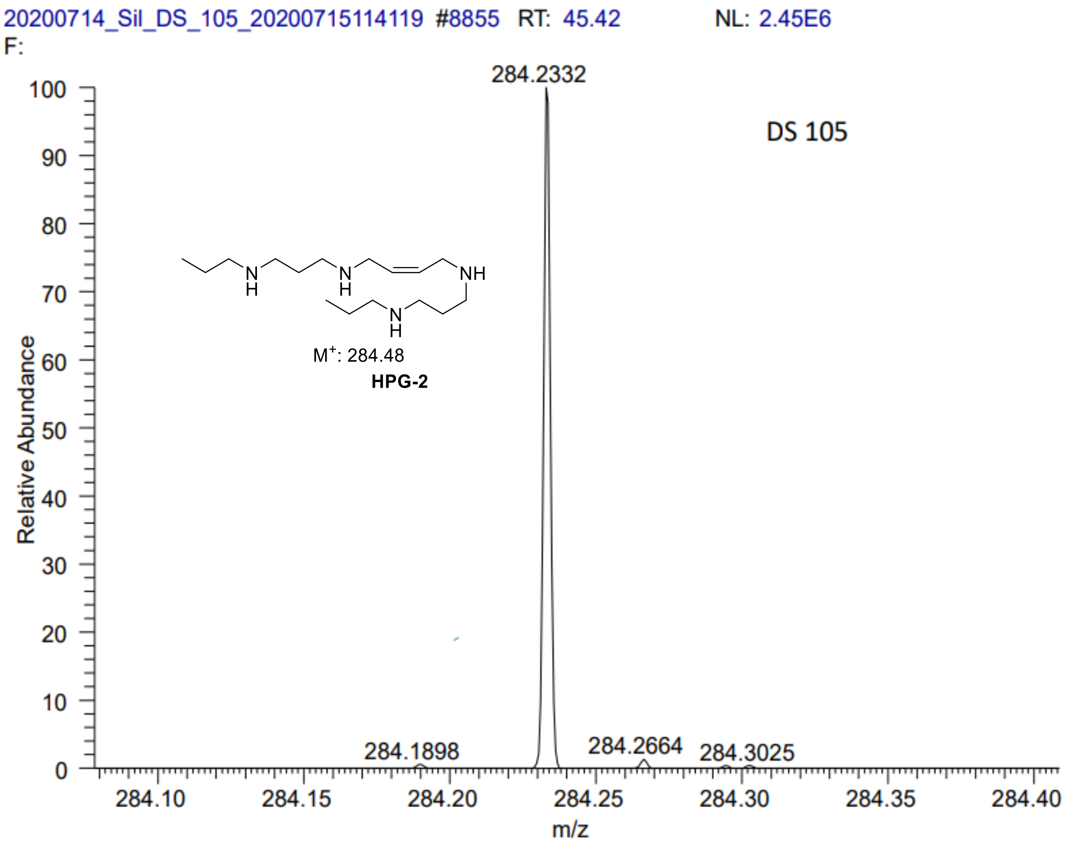

Figure S3. Mass analysis using Orbitrap Fusion of HPG-2

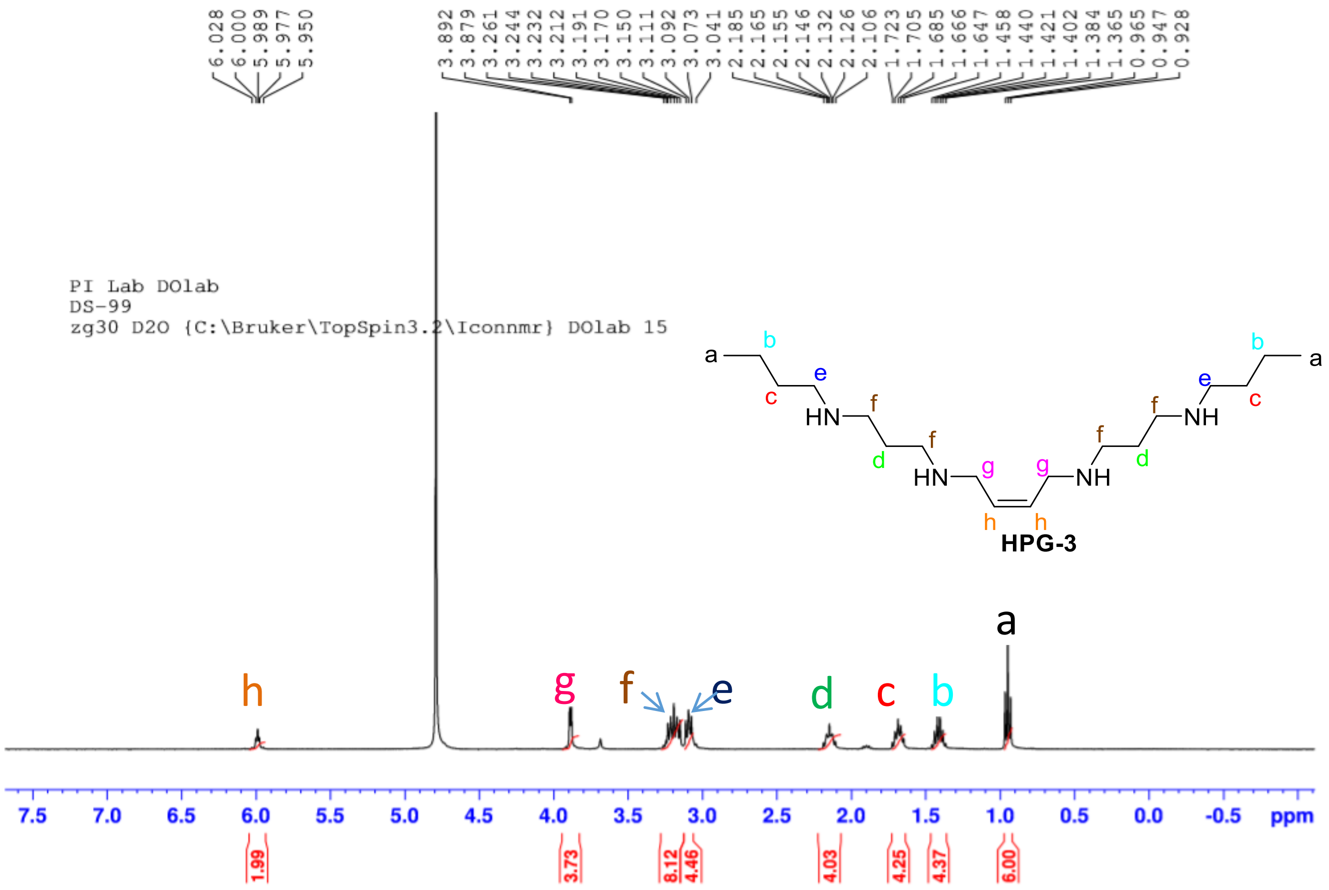

Figure S4. ^{1}H NMR with C_4 side chain (HPG-3)

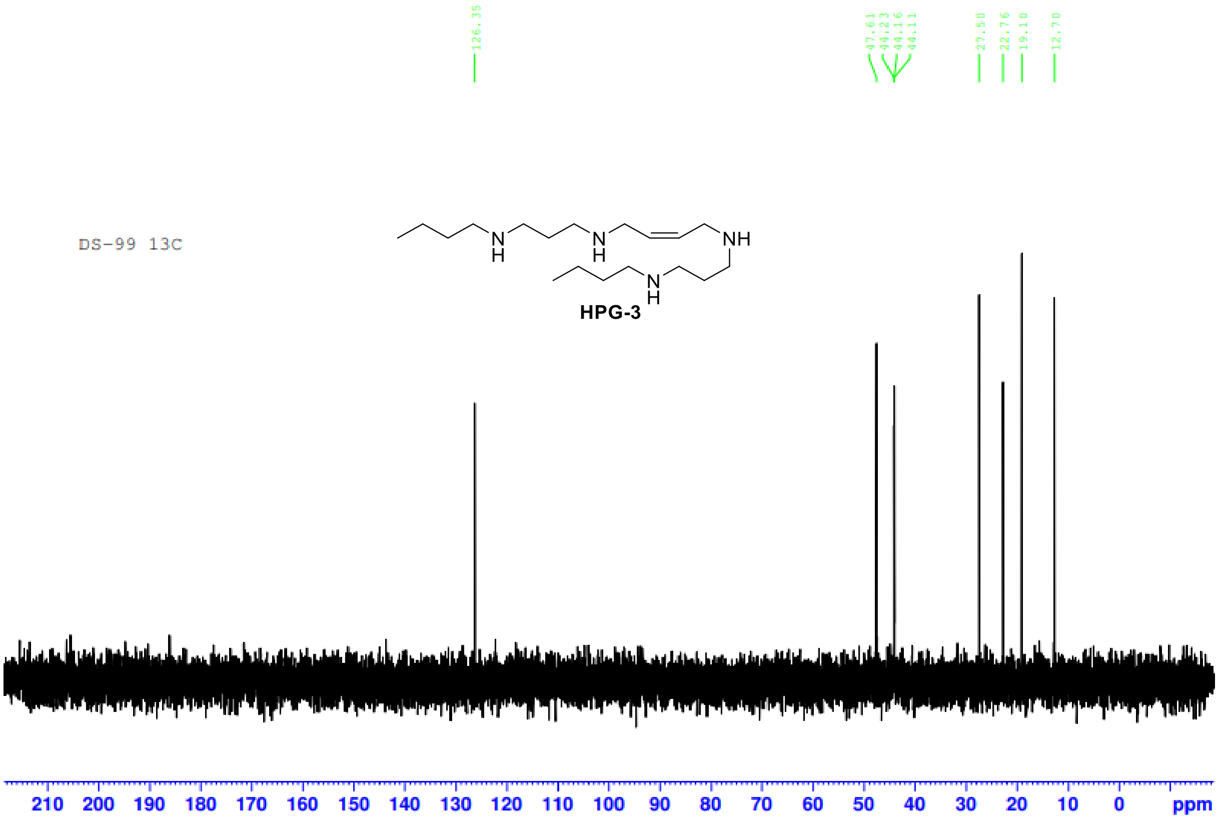

Figure S5. ^{13}C NMR with C_4 side chain (HPG-3)

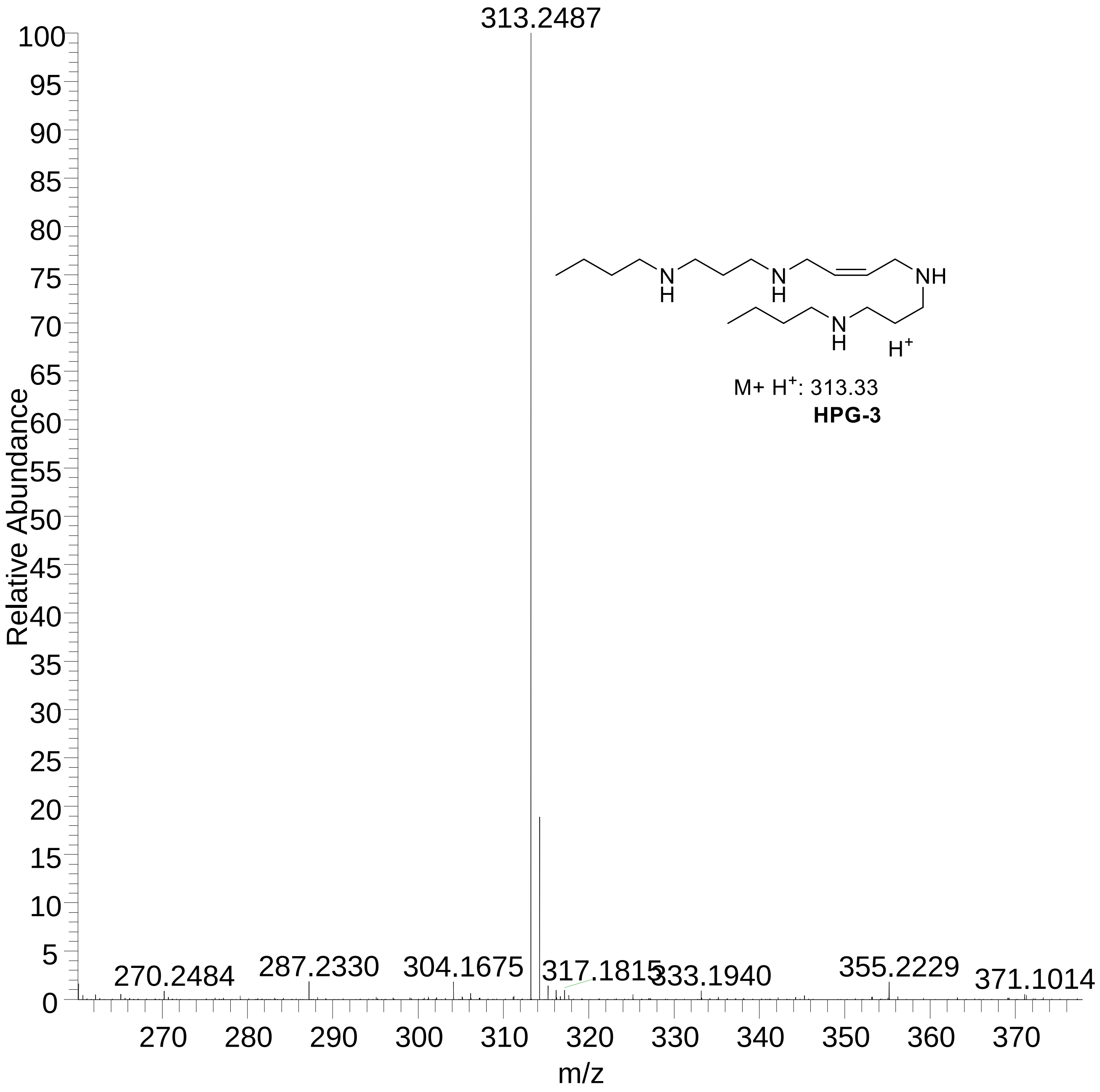

Figure S6. Mass analysis using Orbitrap Fusion of HPG-3

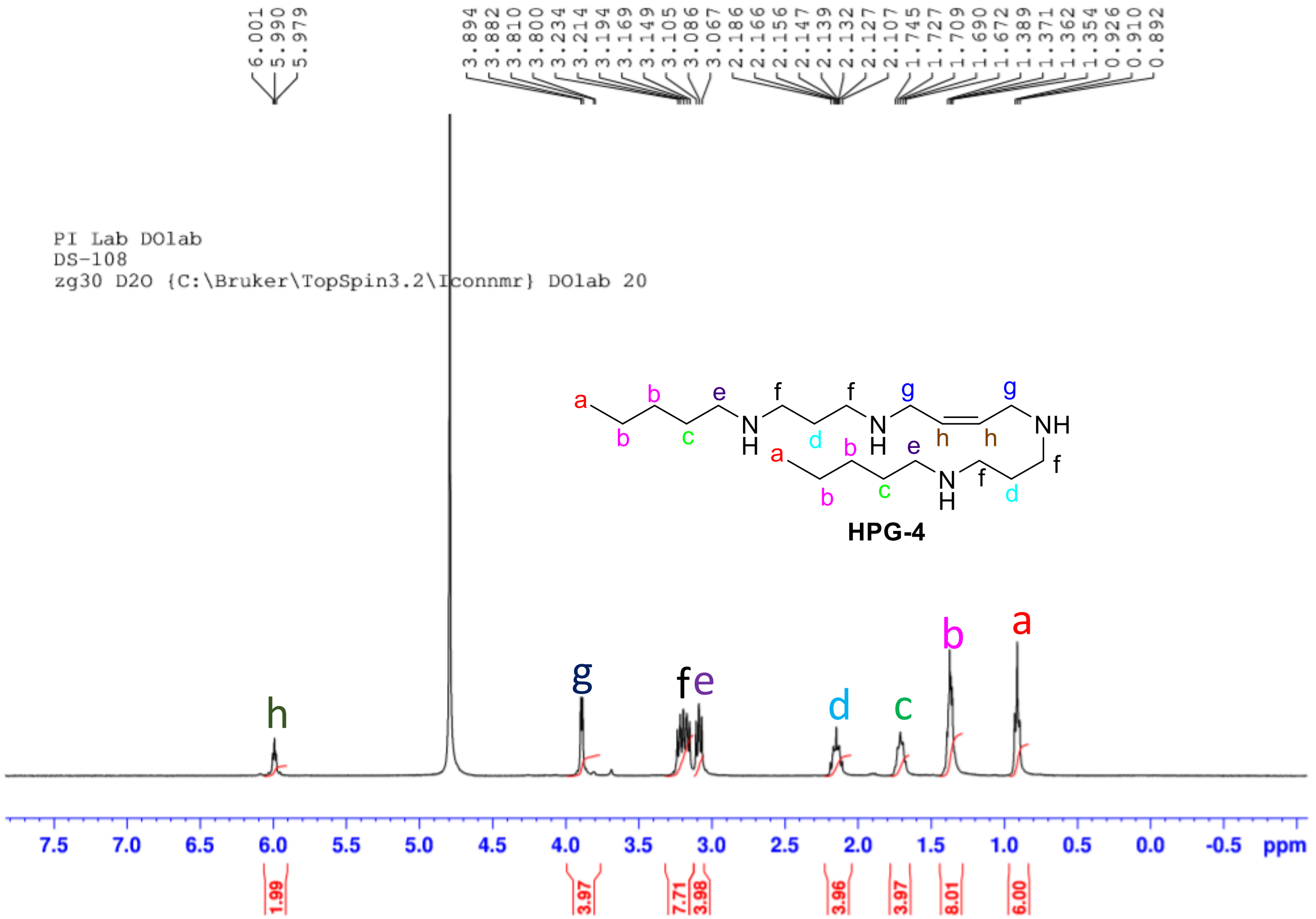

Figure S7. ^{1}H NMR with C_5 side chain (HPG-4)

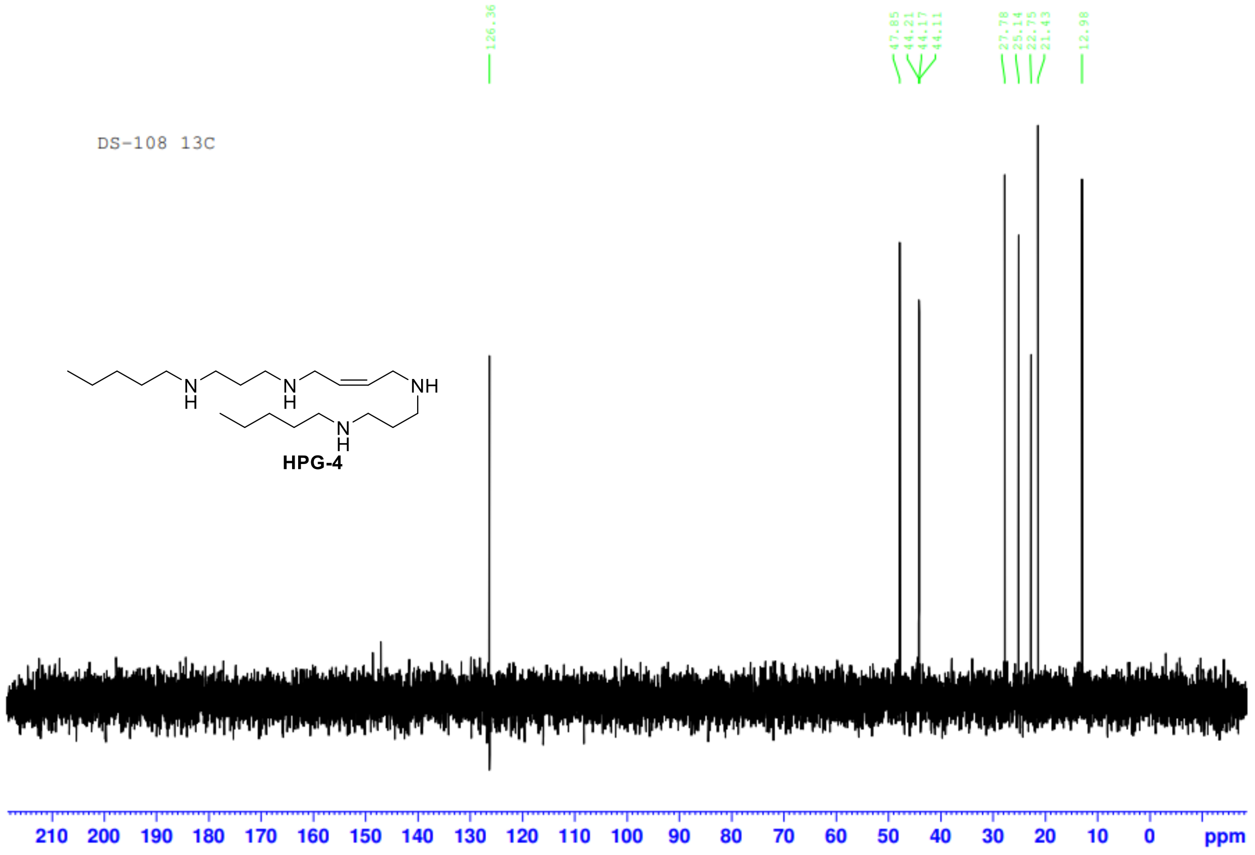

Figure S8. ^{13}C NMR with C_5 side chain (HPG-4)

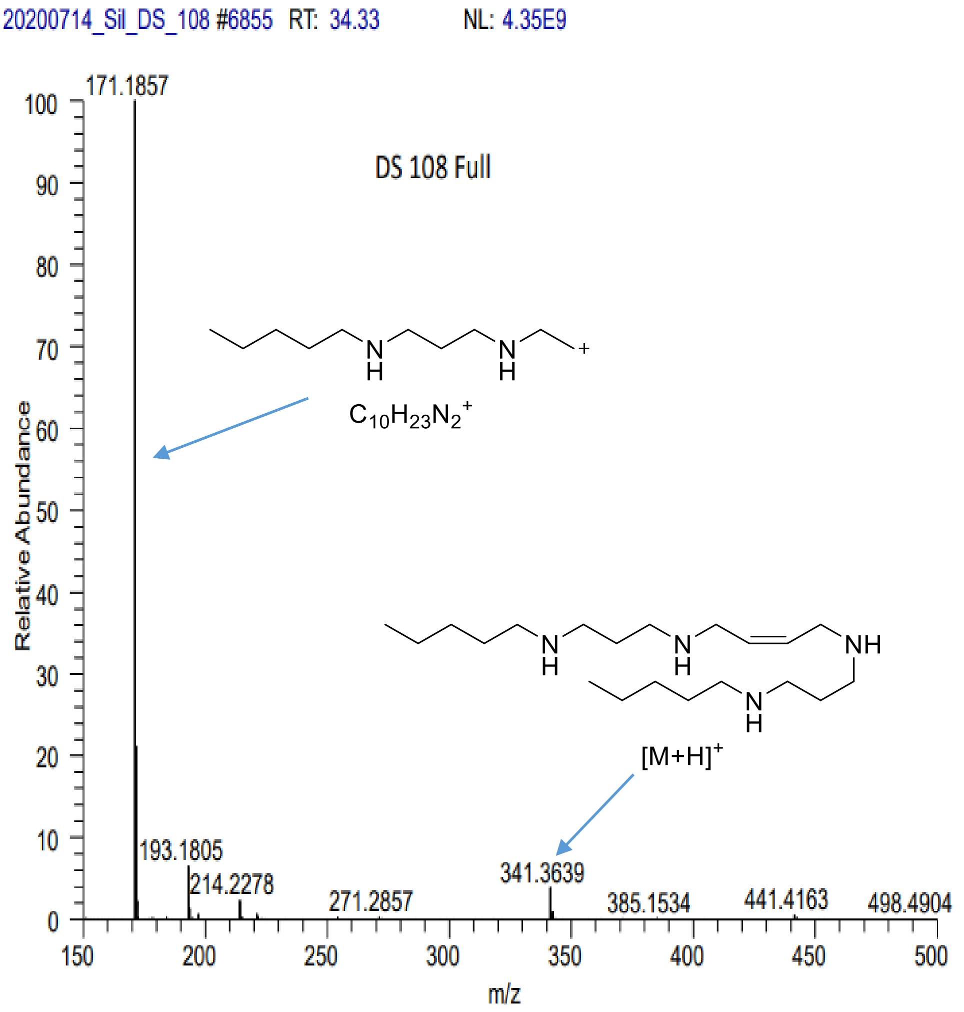

Figure S9. Orbitrap of (HPG-4) (Fragmented product)

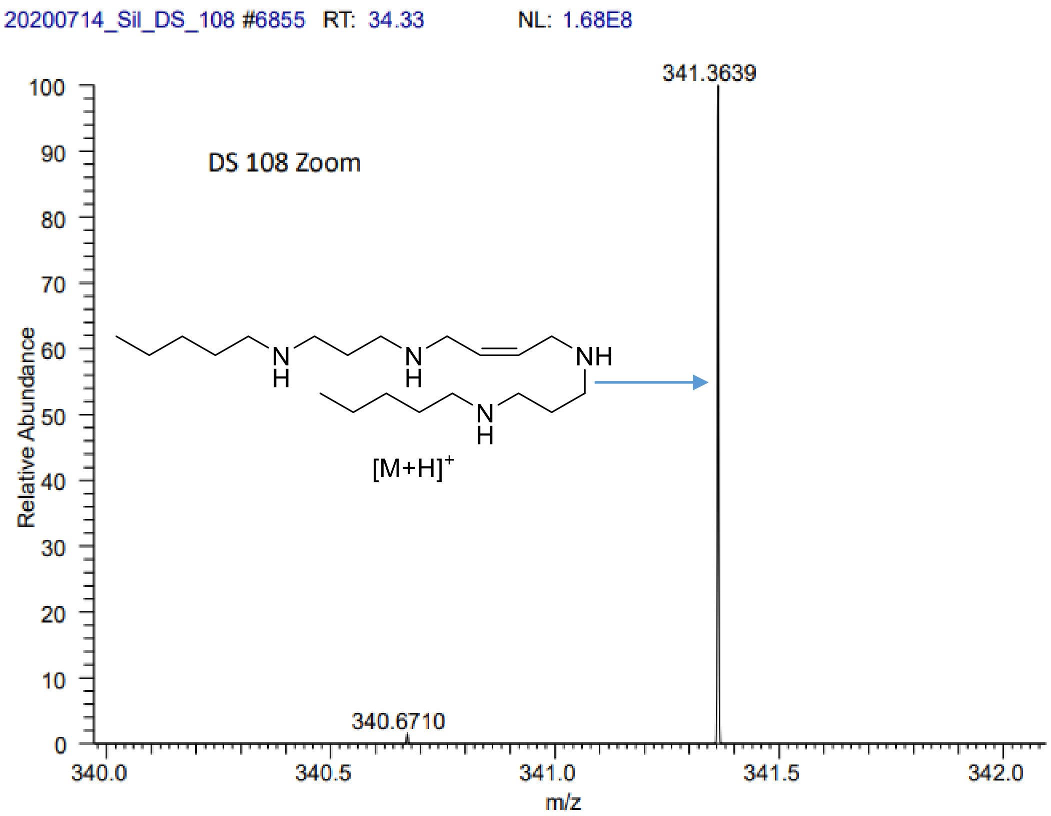

Figure S10. Orbitrap of (HPG-4)

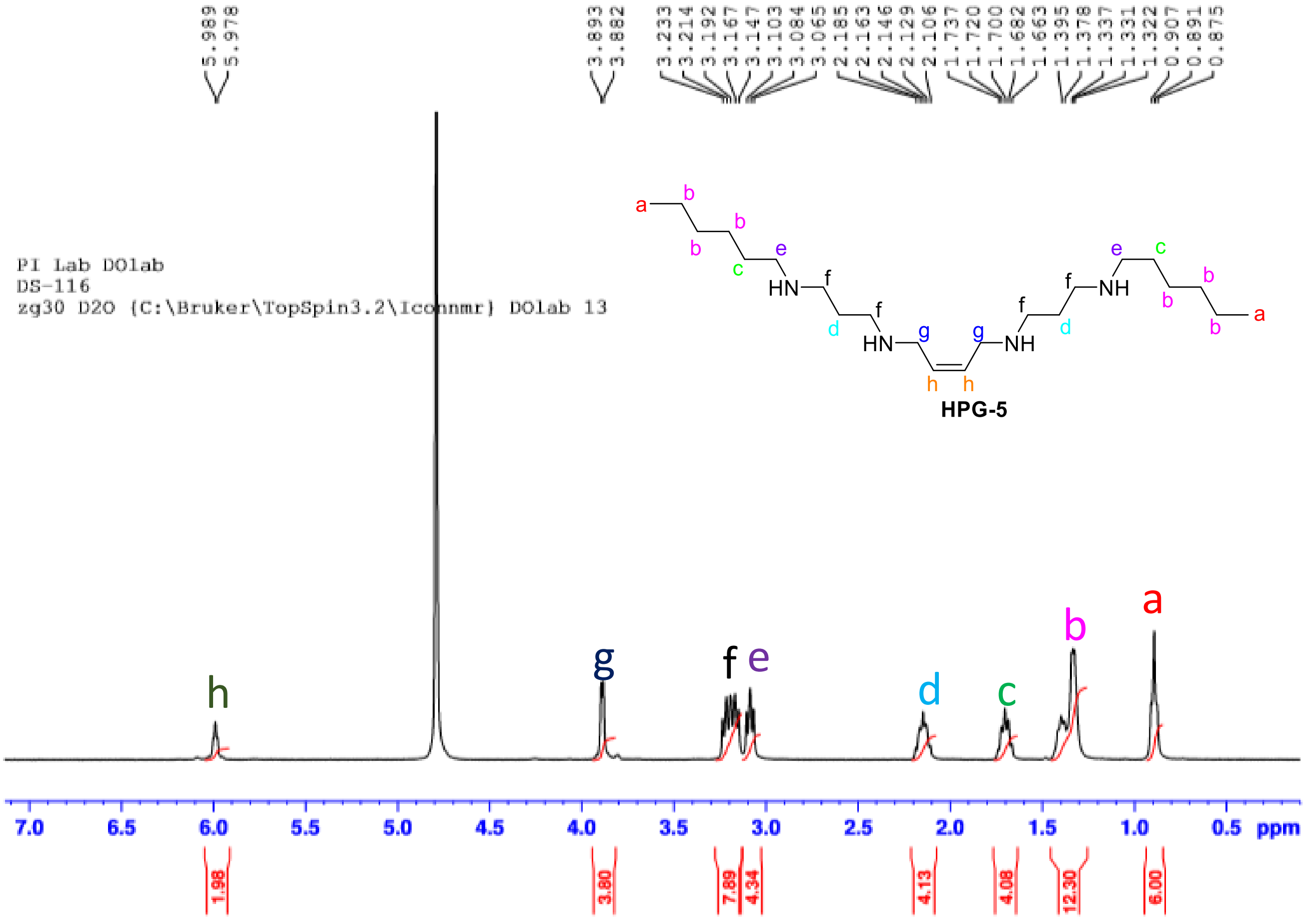

Figure S11. ^{1}H NMR with C_6 side chain (HPG-5)

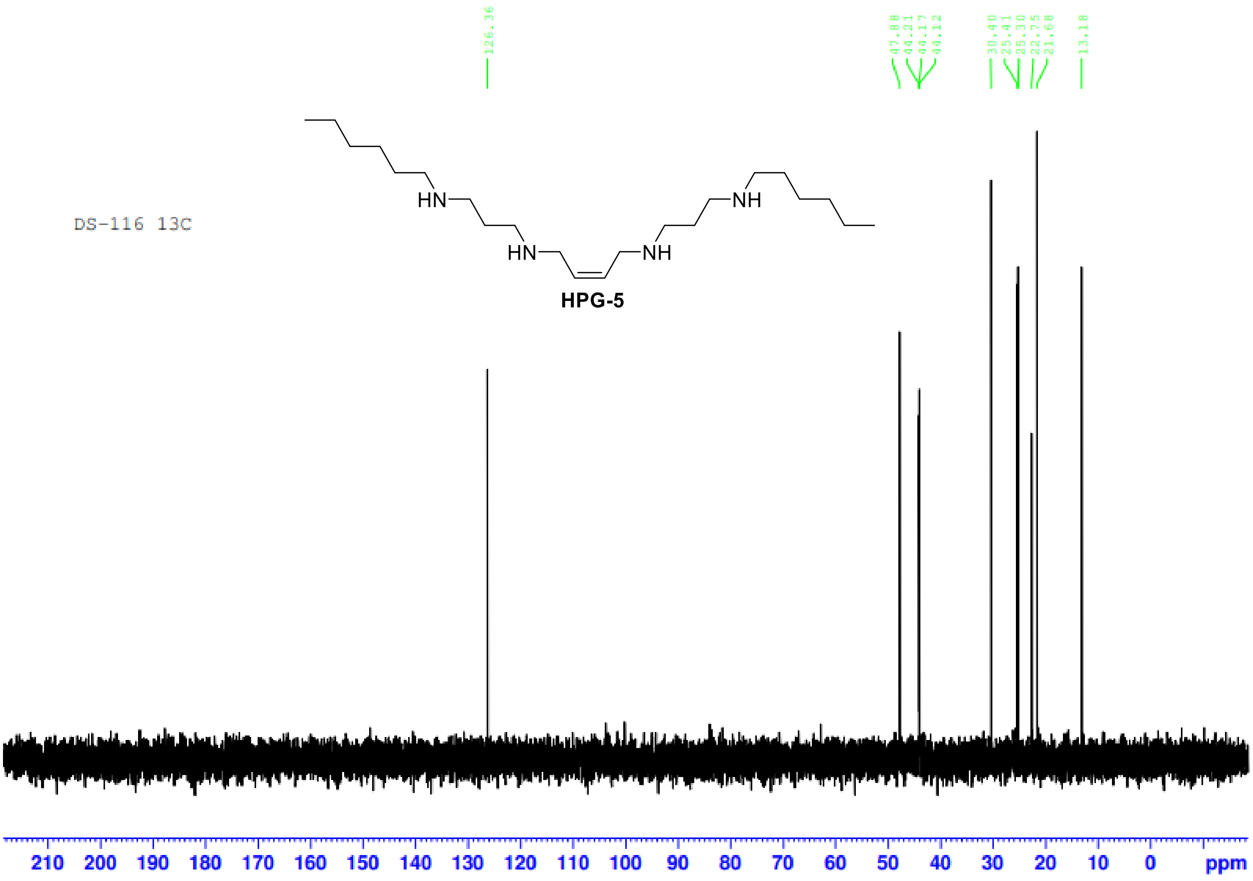

Figure S12. ^{13}H NMR with C_6 side chain (HPG-5)

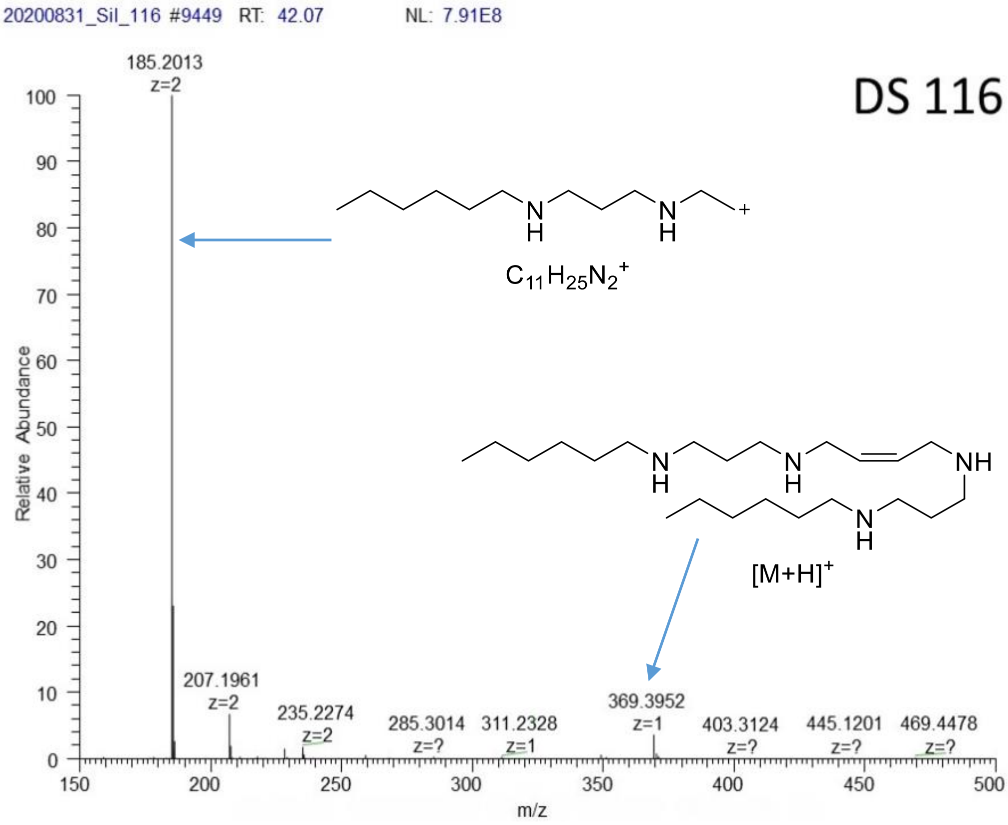

Figure S13. Orbitrap of (HPG-5) (Fragmented product)

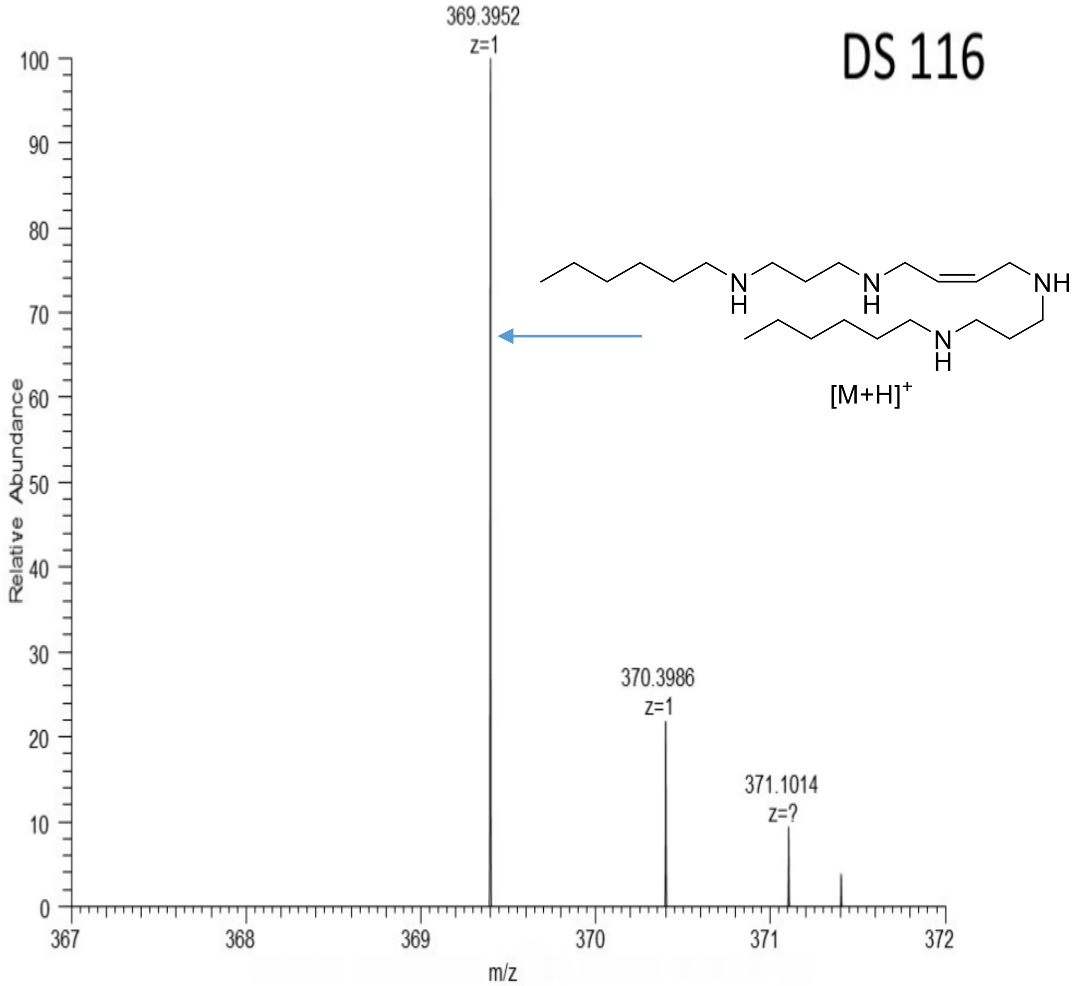

Figure S14. Orbitrap of (HPG-5)

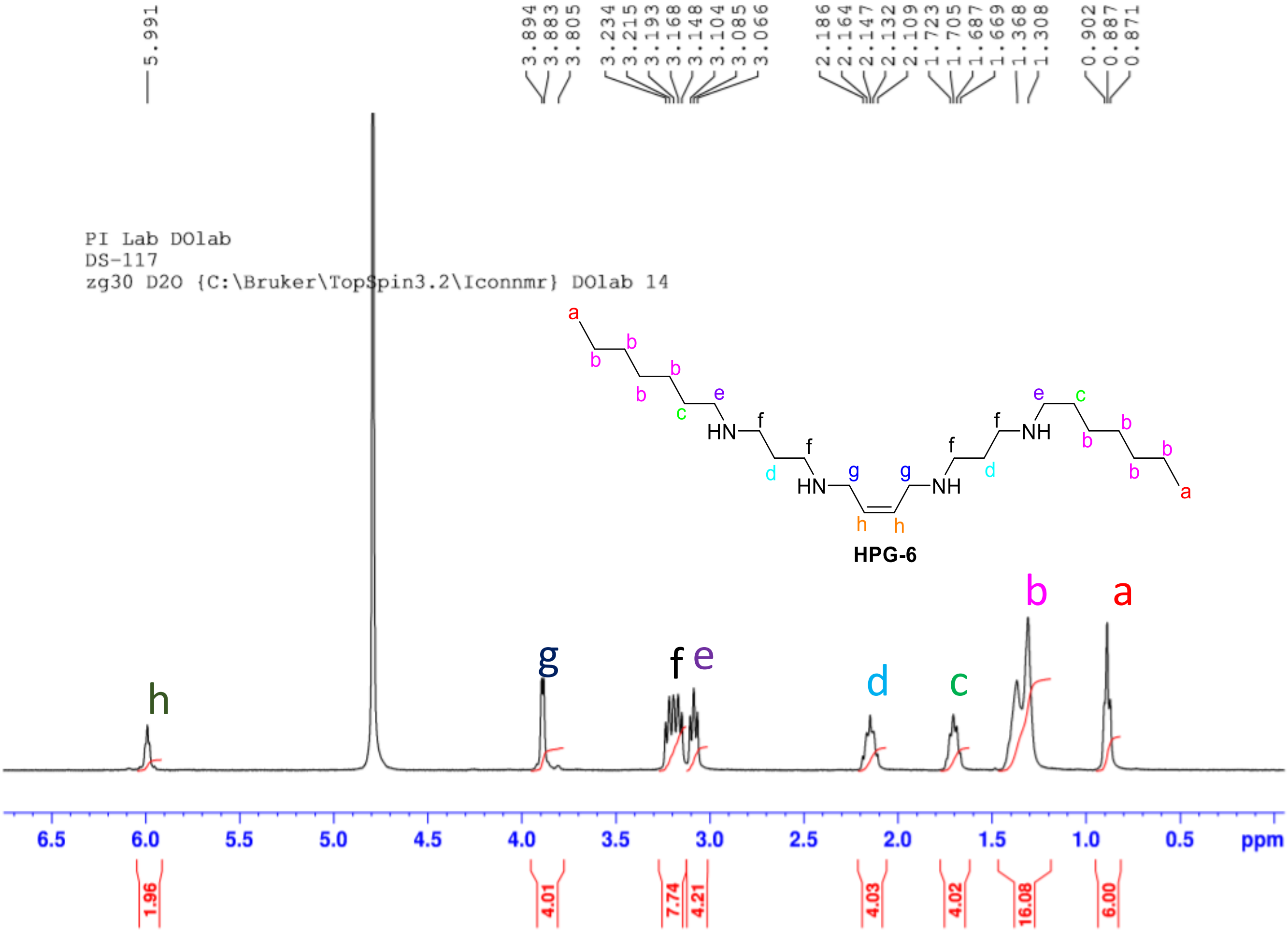

Figure S15. ^{1}H NMR with C_7 side chain (HPG-6)

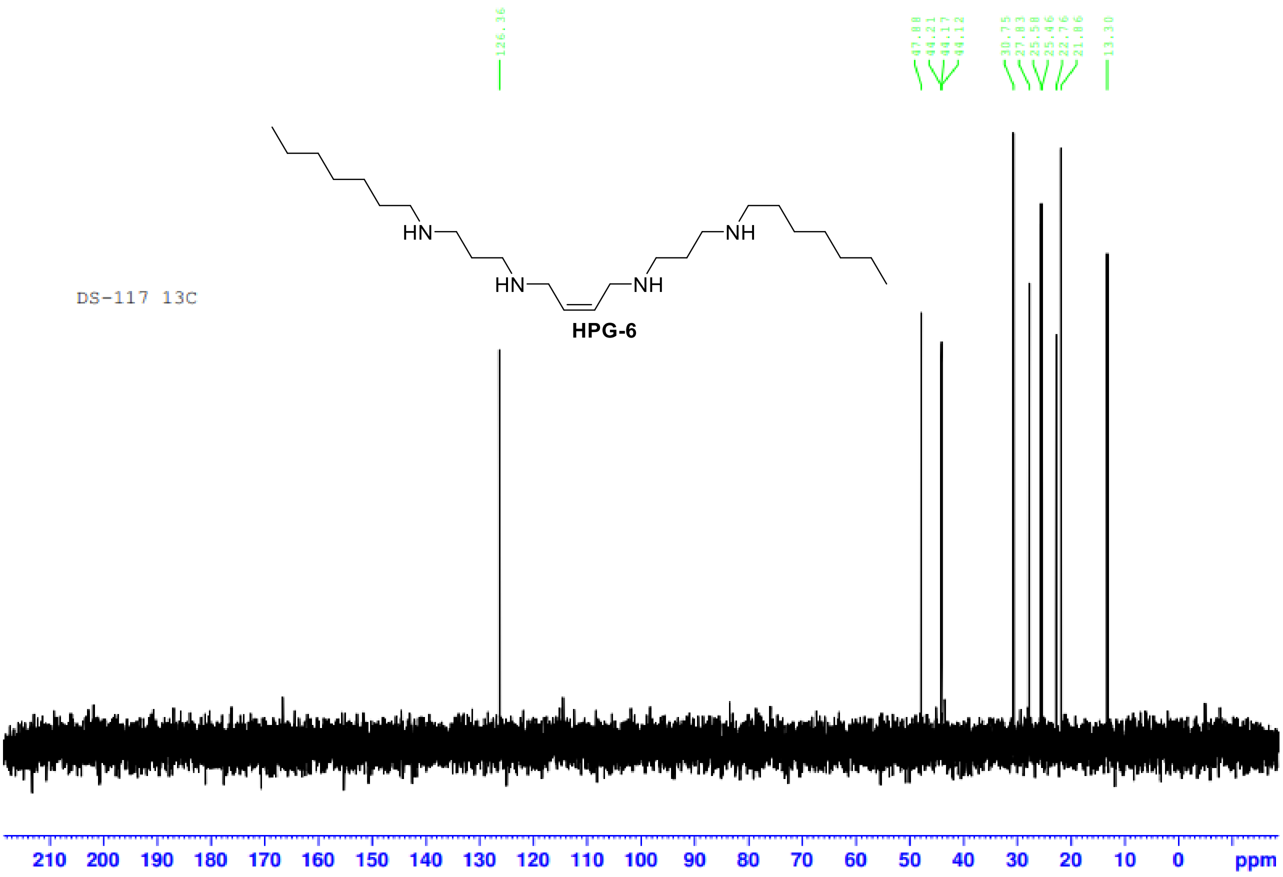

Figure S16. ^{13}C NMR with C_7 side chain (HPG-6)

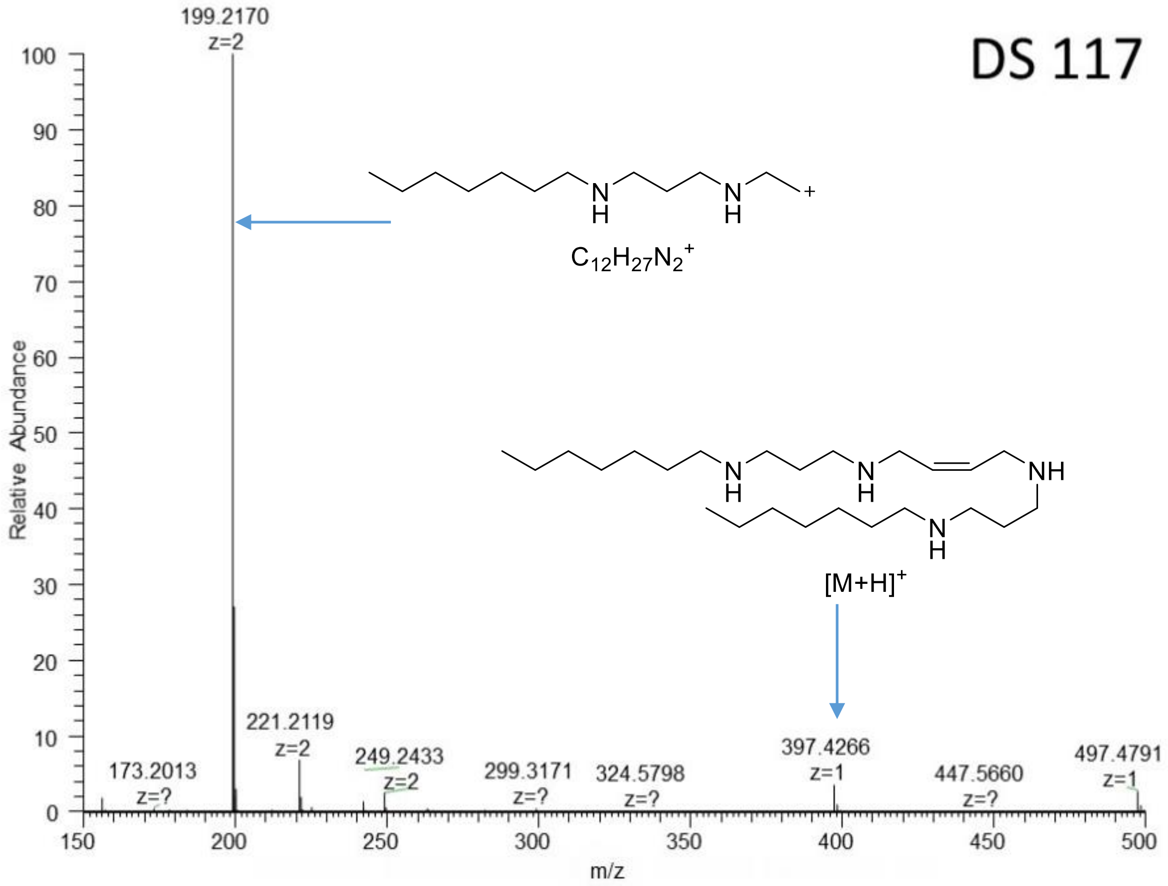

Figure S17. Orbitrap of (HPG-6) (Fragmented product)

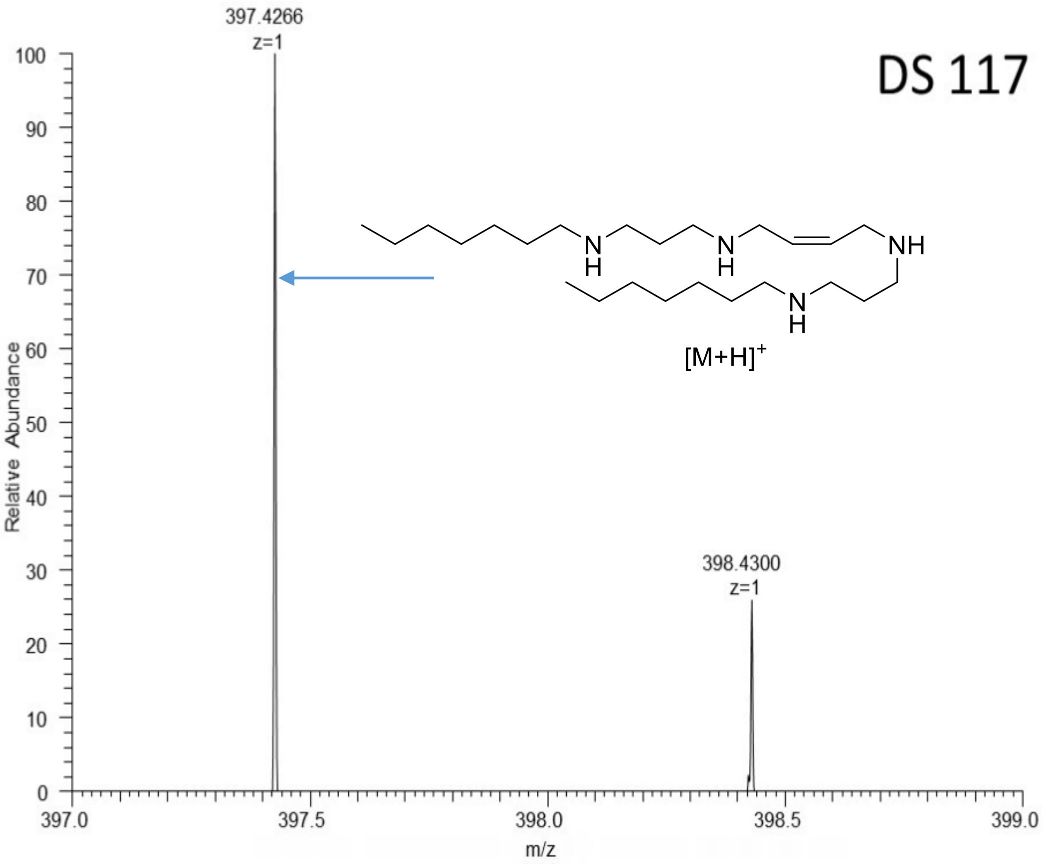

Figure S18. Orbitrap of (HPG-6)

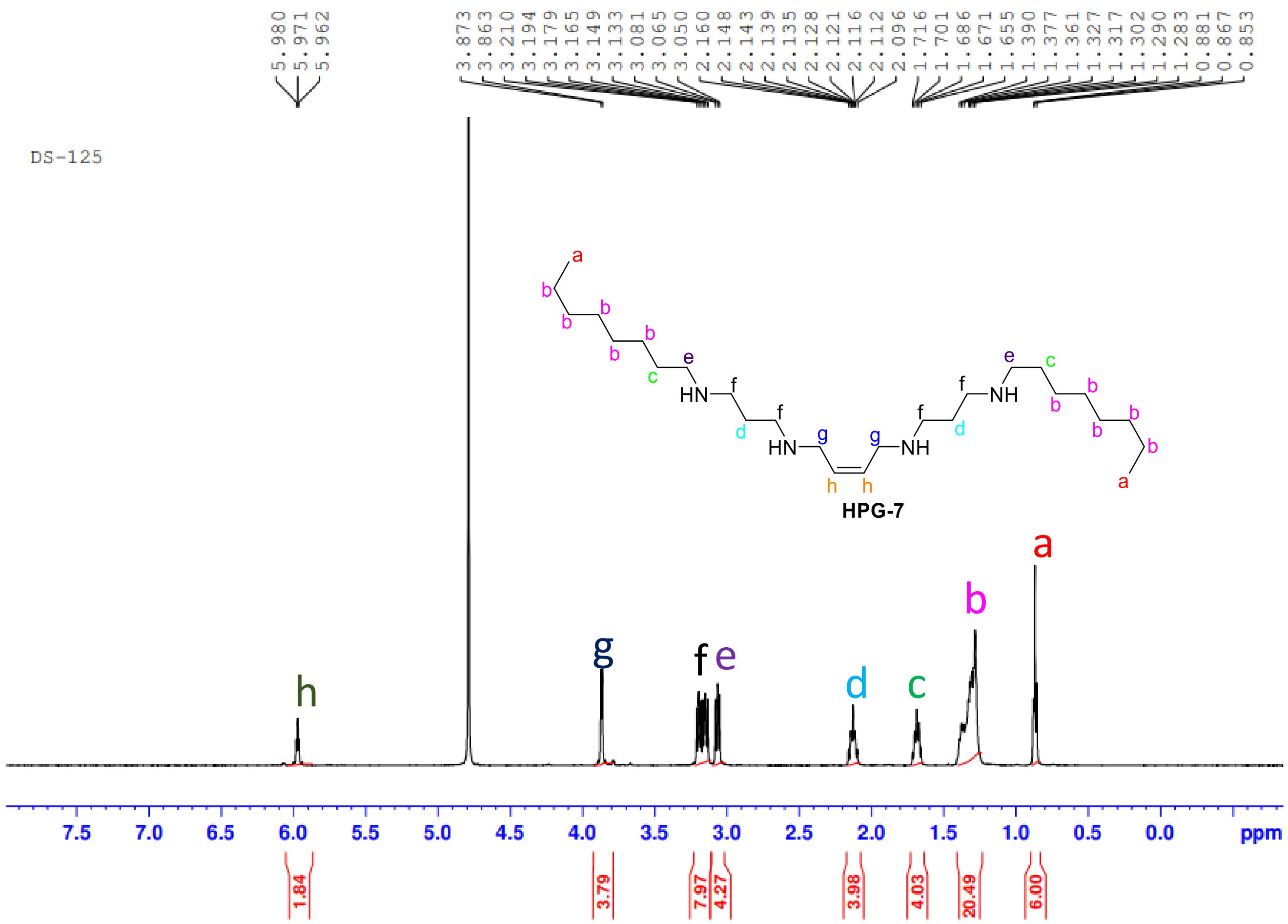

Figure S19. ^{1}H NMR with C_8 side chain (HPG-7)

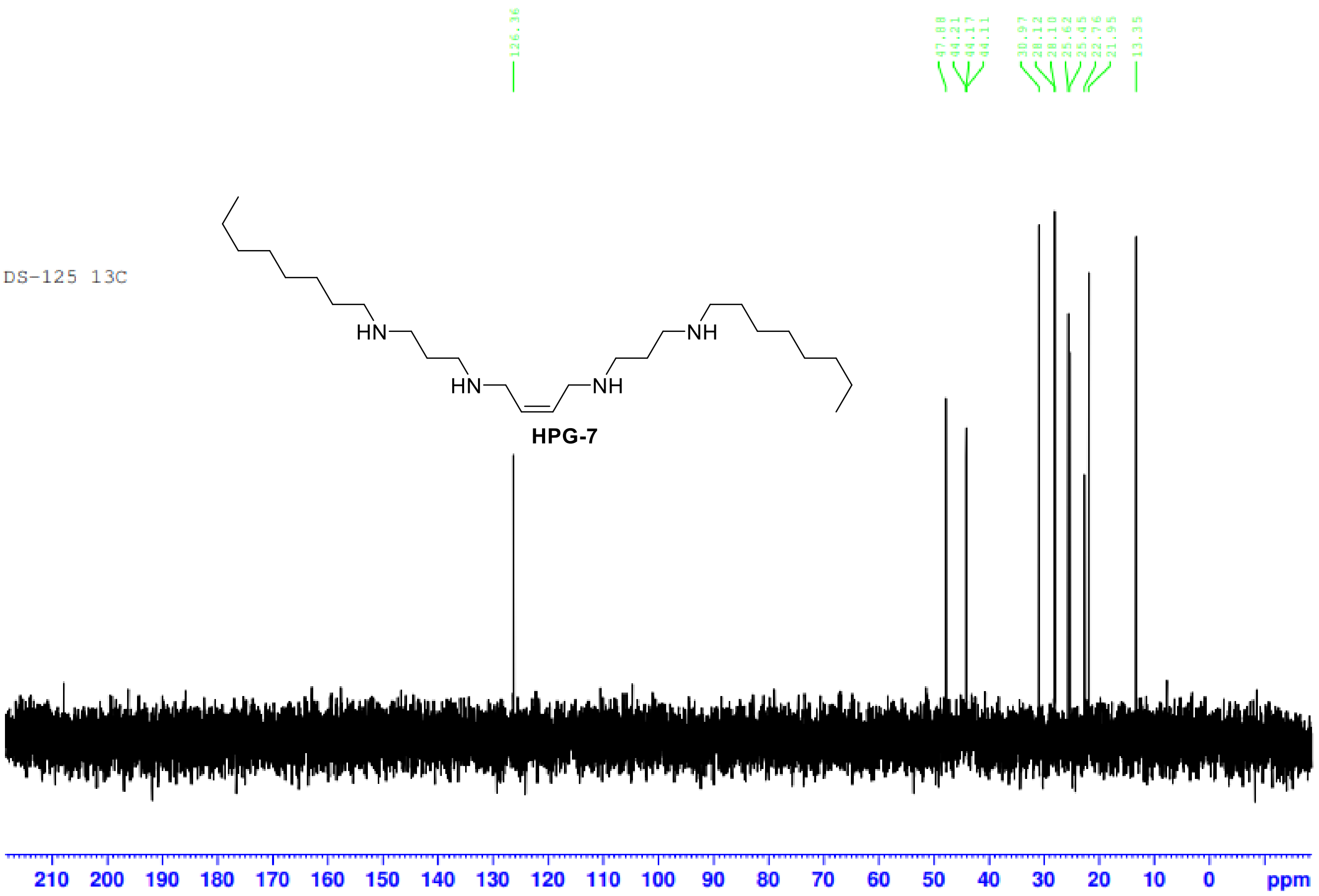

Figure S20. ^{13}C NMR with C_8 side chain (HPG-7)

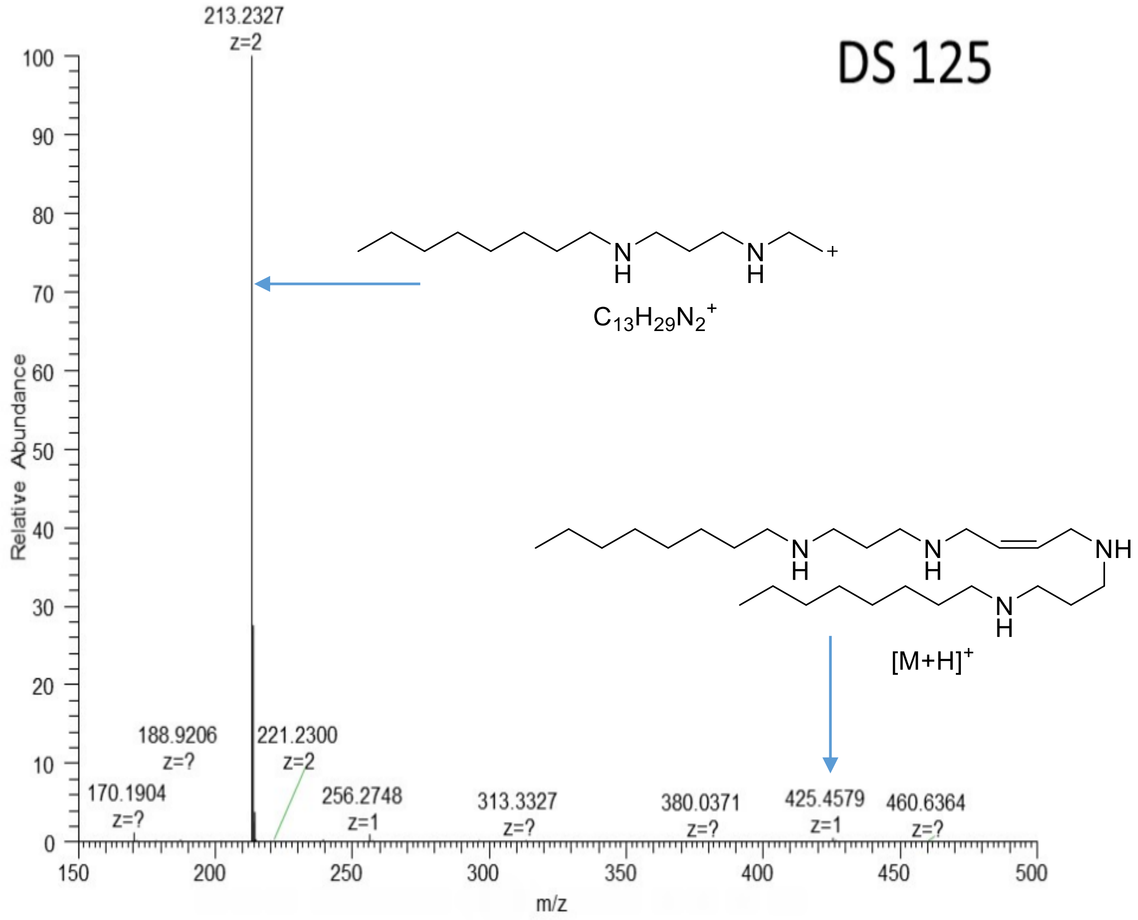

Figure S21. Orbitrap of (HPG-7) (Fragmented product)

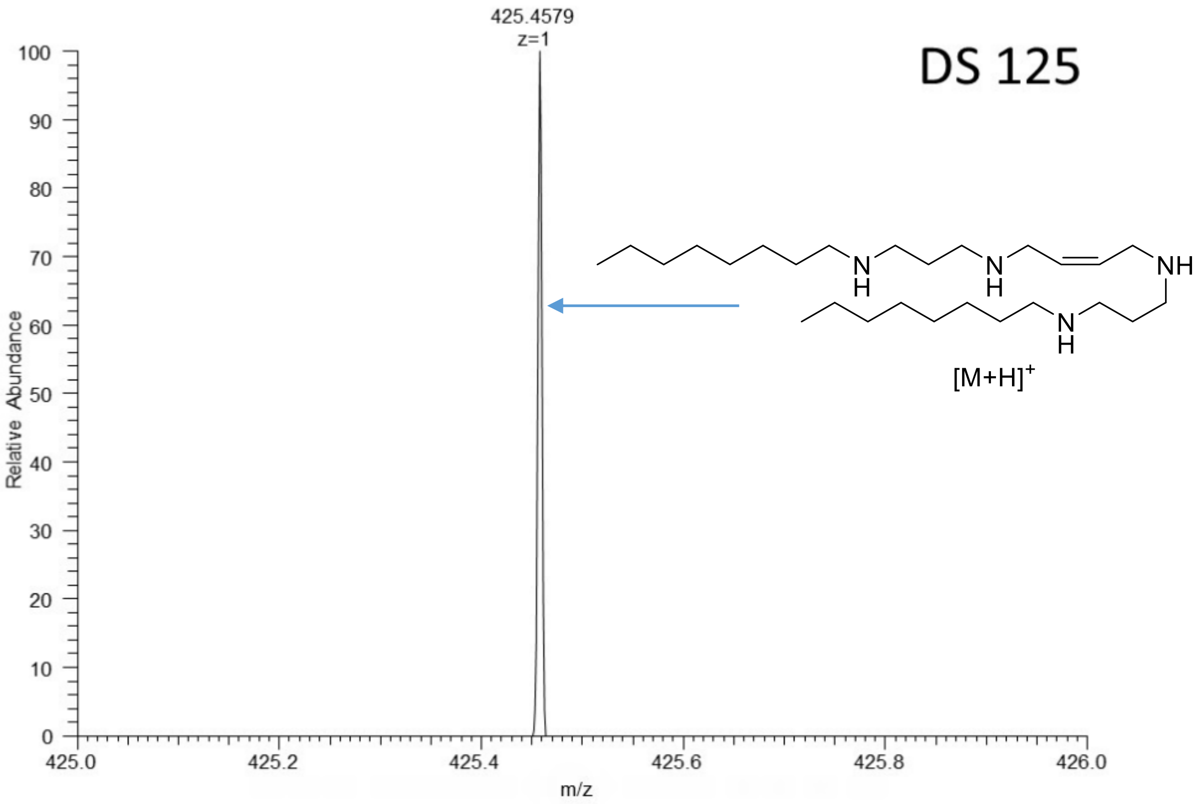

Figure S22. Orbitrap of (HPG-7)

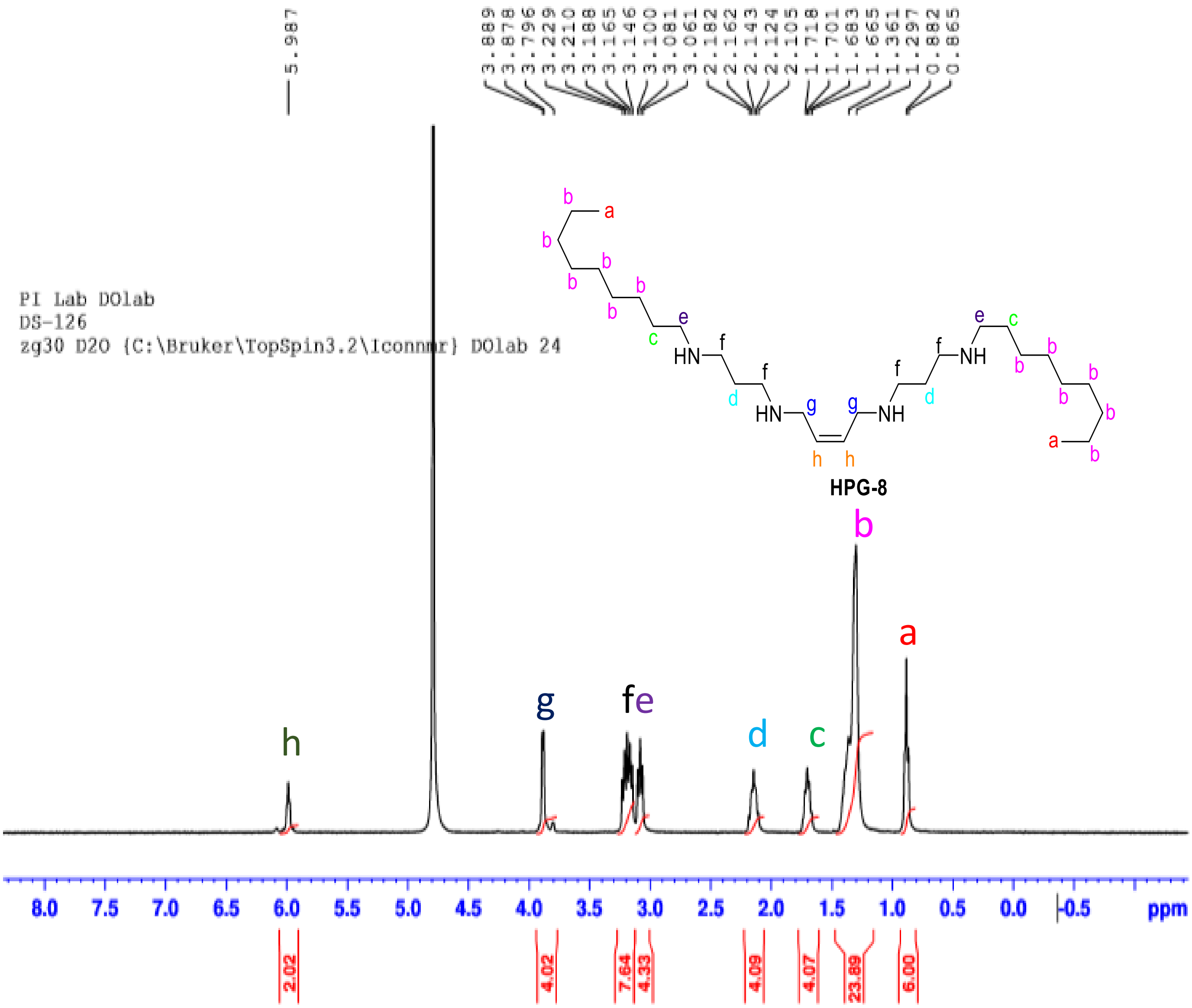

Figure S23. ^{1}H NMR with C_9 side chain (HPG-8)

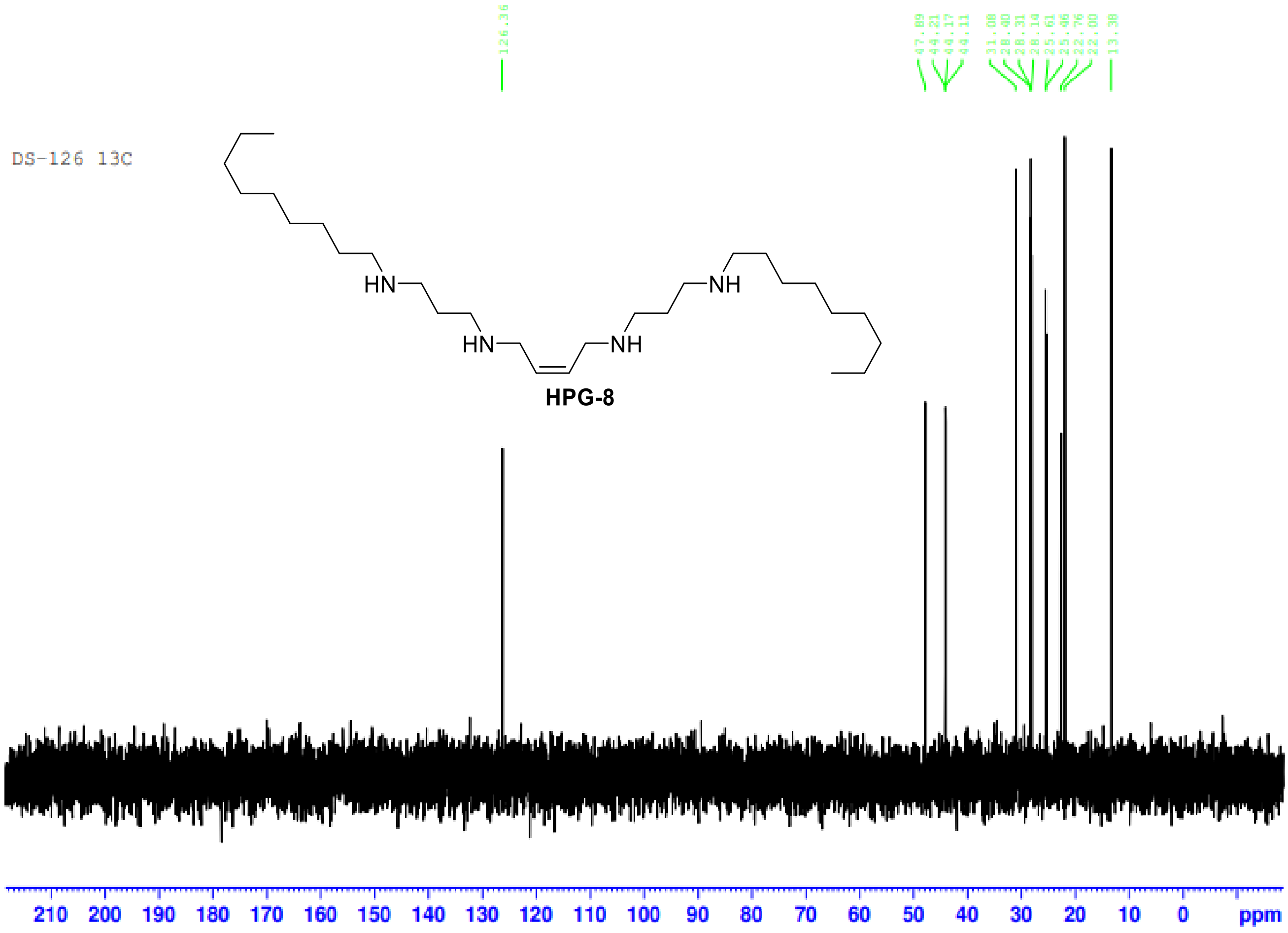

Figure S24. ^{13}C NMR with C_9 side chain (HPG-8)

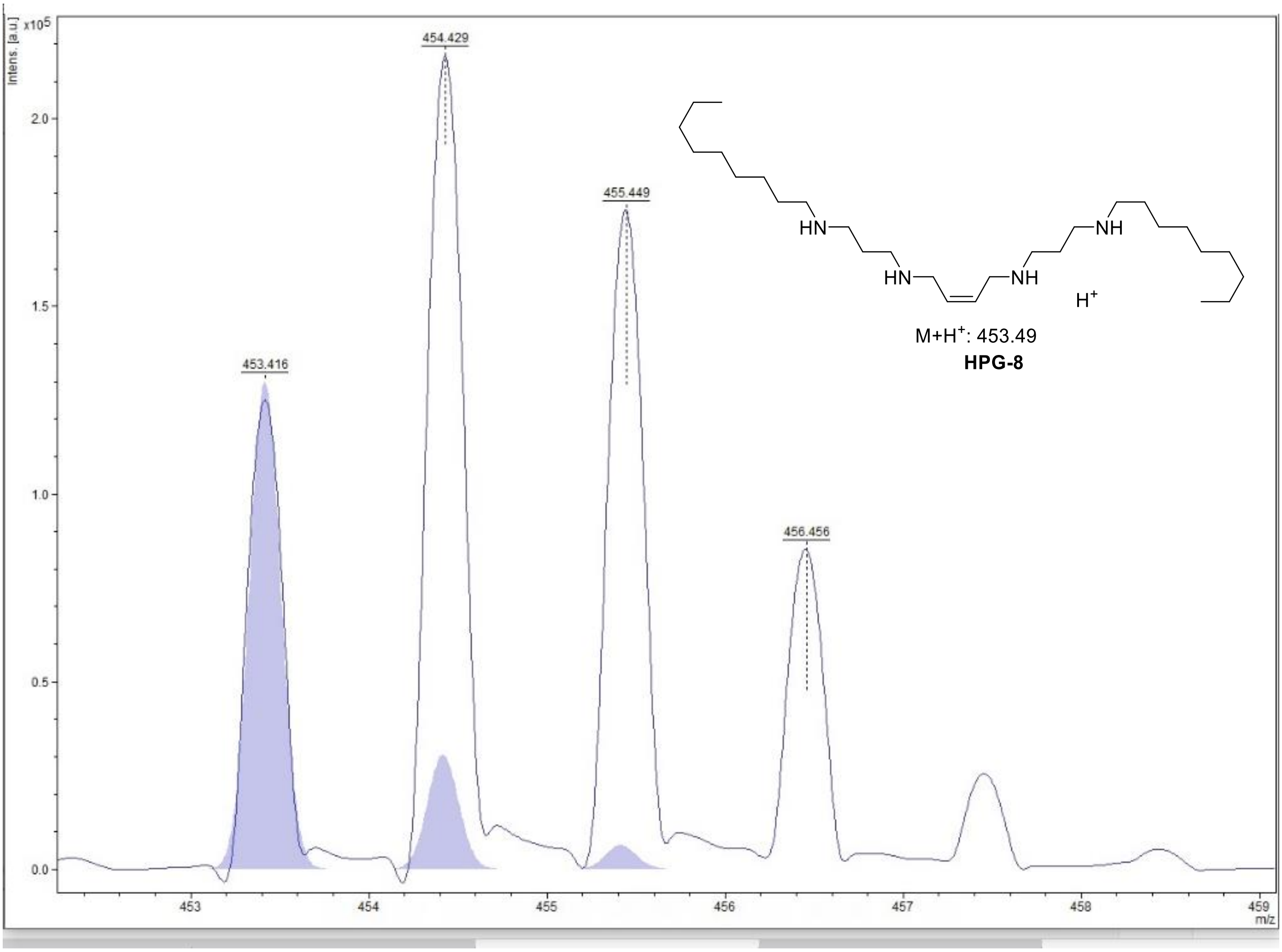

Figure S25. Mass analysis using MALDI of (HPG-8)
